# Role of Ultrafast Internal Conversion and Intersystem Crossing in the Nonadiabatic Relaxation Dynamics of *ortho*-Nitrobenzaldehyde

**DOI:** 10.1021/acs.jpca.3c02899

**Published:** 2023-07-05

**Authors:** Dóra Vörös, Sebastian Mai

**Affiliations:** † Institute of Theoretical Chemistry, Faculty of Chemistry, University of Vienna, Währinger Straße 17, 1090 Vienna, Austria; ‡ Vienna Doctoral School in Physics, University of Vienna, Boltzmanngasse 5, 1090 Vienna, Austria

## Abstract

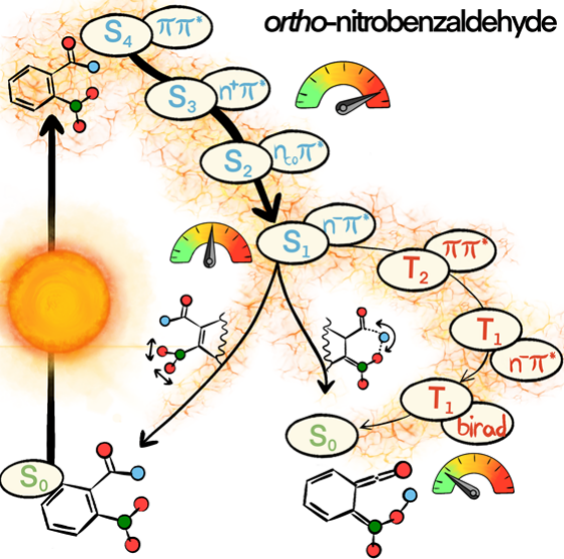

*ortho*-Nitrobenzaldehyde (*o*NBA) is a well-known photoactivated acid and a prototypical photolabile nitro-aromatic compound. Despite extensive investigations, the ultrafast relaxation dynamics of *o*NBA is still not properly understood, especially concerning the role of the triplet states. In this work, we provide an in-depth picture of this dynamics by combining single- and multireference electronic structure methods with potential energy surface exploration and nonadiabatic dynamics simulations using the Surface Hopping including ARbitary Couplings (SHARC) approach. Our results reveal that the initial decay from the bright ππ* state to the S_1_ minimum is barrierless. It involves three changes in electronic structure from ππ* (ring) to *n*π* (nitro group), to *n*π* (aldehyde group), and then to another *n*π* (nitro group). The decay of the ππ* takes 60–80 fs and can be tracked with time-resolved luminescence spectroscopy, where we predict for the first time a short-lived coherence of the luminescence energy with a 25 fs period. Intersystem crossing can occur already during the S_4_ → S_1_ deactivation cascade but also from S_1_, with a time constant of about 2.4 ps and such that first a triplet ππ* state localized on the nitro group is populated. The triplet population first evolves into an *n*π* and then quickly undergoes hydrogen transfer to form a biradical intermediate, from where the ketene is eventually produced. The majority of the excited population decays from S_1_ through two conical intersections of equal utilization, a previously unreported one involving a scissoring motion of the nitro group that leads back to the *o*NBA ground state and the one involving hydrogen transfer that leads to the ketene intermediate.

## Introduction

The first studies on the rich photochemistry of nitro-aromatic compounds date back to the early 20th century.^1^ Shortly after the discovery of those kinds of photoreactions, comprehensive investigations followed to identify potential applications such as photosensitive protecting groups.^2−4^*ortho*-Nitrobenzaldehyde (*o*NBA) is one of these early-discovered photosensitive nitro-aromatic molecules, acting as a photoactivated acid. This characteristic of *o*NBA is commonly applied, e.g., in different pH-controlled processes,^5−8^ as a photoactivatable proton source.

The photoisomerization of *o*NBA to nitrosobenzoic acid occurs through a γ-hydrogen abstraction,^9,10^ leading to a ketene intermediate. From this stage, the reaction is irreversible and the photoproduct is formed.^11,12^ The reaction happens with an overall quantum yield of about 50%,^11,13^ which appears to be rather insensitive to the nature of the available solvent.^14^ Early investigations of the fundamental relaxation pathway raised the important question regarding the nature of the photoreactive state:^14−17^ Is the ketene formed via triplet or singlet excited states? Further investigations^16,17^ suggested that the singlet excited state mechanism is the key pathway for the photorearrangement. However, these investigations did not exclude the presence of a triplet excited state, which is either formed in low yield or happens to be short-lived (less than 35 ps).

As spectroscopic techniques and quantum chemistry programs improved, it became possible to observe the ultrafast kinetic components and obtain additional insights into the early events in the relaxation mechanism of *o*NBA.^12,18−26^ The application of fluorescence spectroscopy^19,21^ enabled detection of two important time constants. Initially, a significant amount of the fluorescence signal decays within 100 fs (τ_1_), which was assigned to the relaxation from the ^1^ππ* to lower-lying dark ^1^*n*π* states via internal conversion (IC). The second time constant in the red wing of the signal (τ_2_ = ∼400 fs)^19^ τ_2_ was assigned to the depletion of the *n*π* states, which was suggested to occur through three different deactivation channels. First, the excited wave packet could reach a conical intersection (CI) between the ground state and the first excited state to nonradiatively relax back to the ground state with a quantum yield of approximately 50%.^20^ Second, the molecule can relax to the ketene ground state.^20,27^ The signal of the ketene intermediate in the infrared (IR) experiments^20^ is observed within a few 100 fs, which indicates that a hot ketene is formed rapidly. Subsequently, during vibrational cooling, the IR band of the ketene adopts a room-temperature position and width on a 10 ps time scale (τ_3_).^20,21,28^ Third, another decay channel was found, the delayed rise of the ketene intermediate (about one-third of the ketene yield) with a time constant of 220 ps (τ_4_),^27^ much longer than vibrational cooling. The phosphorescence spectrum^29^ of *o*NBA provides a direct evidence of the presence of triplet states; therefore, this third channel was suggested to involve intersystem crossing (ISC) to the triplet manifold. Based on τ_4_ and on the assumption that the ketene is formed in a high yield from the triplet states, the ISC rate constant^27^ was estimated to be *k*_ISC_ = (2.7 ps)^−1^, comparable with that found in nitrobenzene.^30,31^ The ISC is expected^27^ to take place from a low-lying ^1^*n*π* state. Thus, based on El-Sayed’s rule,^32^ the populated triplet state is expected to have ^3^ππ* character. However, earlier studies on nitrobenzene derivates showed that the lowest triplet state has an *n*π* character. Consequently, ISC should be followed by IC to lower triplet state(s) ().^21^

**Figure 1:**
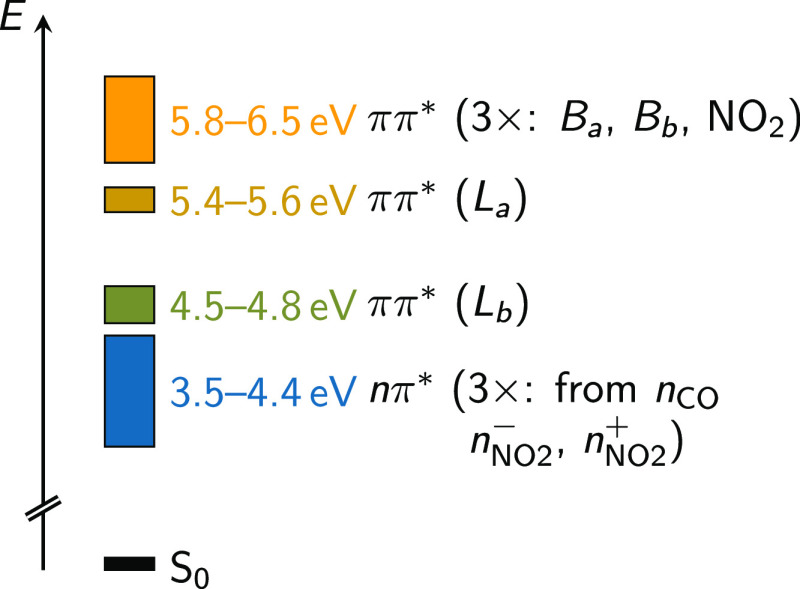
Relevant low-lying and bright singlet excited states of *o*NBA according to ref (33).

Several theoretical investigations^12,22−25^ in 2008–2011 shed some light on the underlying electronic structure and on possible singlet relaxation pathways of *o*NBA. Basic assignment of excitation characters of the experimental absorption spectrum was performed by Leyva et al.^22,24^ with excited state calculations using several different electronic structure methods. More recently, we^33^ provided a more comprehensive investigation of the vertically excited states of *o*NBA using several correlated single-reference methods and complete active space second-order perturbation theory (CASPT2), aided by extensive state-of-the-art wave function analysis. The broad tail of the *o*NBA spectrum—around 300 nm—is due to several *n*π* excitations localized on the carbonyl and nitro groups. The higher-energy region (270–240 nm) is composed mainly of states with ππ* and charge-transfer (CT) contributions. A schematic overview of the vertical positions of these states^33^ is provided in Figure 1 (see the beginning of the Results and Discussion Section for a discussion of these states as well as the triplets).

Leyva et al.^12^ investigated two possible deactivation channels that could occur within the singlet manifold after excitation into one of the bright ππ*/CT states. The dominant path involves sequential IC, starting from either the ππ* (*L*_*a*_) or ππ* (*L*_*b*_) state (S_5_ or S_4_, respectively, according to ref (33))—depending on the initial excitation wavelength—and ending in the minimum of the S_1_ (lowest-lying *n*π* state localized on the nitro group^33^). Subsequently, an S_1_/S_0_ crossing with a long carbonyl C–H bond length can be reached, preparing the hydrogen transfer (HT) reaction, driven by the hole in the nitro lone pair orbital of the *n*π* state.^22^ From that S_1_/S_0_ crossing point, the molecule can either react back to the *o*NBA ground state or form the ketene intermediate. The second deactivation channel to the ketene intermediate was suggested to start already in the higher excited states (e.g., S_3_) followed by IC along the HT coordinate. For the sake of comparison of the two pathways, the energy barrier for reaching the HT coordinates was computed, leading to the conclusion that the main pathway includes first the IC cascade and subsequently the ketene formation from the lowest-lying excited state. However, Leyva et al.^12^ did not include the triplet states in the proposed deactivation channels, instead assuming that the second channel competes with the population of the triplet manifold.

In the present study, we aim at answering several relevant questions about the *o*NBA photorelaxation that are still open. Regarding the earliest phase of the dynamics, we scrutinize how long-lived the initially excited ππ* states are and whether the system first relaxes to one of the *n*π* states or activates the HT/ketene formation process already before. Here, we also clearly assign the experimental 50–100 fs time constant (τ_1_) obtained from time-resolved fluorescence. We then investigate the fate of the *n*π* (S_1_) gateway state, scrutinizing which deactivation channels exist, their relative importance, their corresponding products, and whether they are specific to *o*NBA or are inherited from parent compounds. Moreover, we analyze the role of the triplet states and investigate whether ISC occurs from S_1_ or already from higher-lying states, which electronic characters are involved, how large the ISC yield can be expected to be, and what its time scale is. Additionally, the type of formed triplet state and its possible decay routes and times are revisited. To answer this set of questions, we have explored the potential energy surfaces (PESs) at a high level of theory, including the triplet PESs, in terms of relevant critical geometries. Subsequently, we have performed nonadiabatic dynamics simulation to reinforce our findings and to understand better the time scales, yields, and time-resolved spectra observed after excitation.

## Methods

The computations are divided into two steps. In the first step, minima, minimum-energy conical intersections (MECI), and minimum-energy singlet–triplet crossing points (MECP) were optimized and connected through interpolation scans to obtain an overview of the relevant deactivation pathways. In the second step, we performed nonadiabatic dynamics simulations using the Surface Hopping including ARbitary Couplings (SHARC) package.^34−36^ This step was primarily focused on understanding the initial relaxation from the bright singlet states to the S_1_ and whether ISC could play a role during this relaxation.

The calculations of the first step were performed side-by-side with the multistate complete active space second-order perturbation theory (MS-CASPT2)^37,38^ and algebraic diagrammatic construction scheme to second order for the polarization propagator (ADC(2))^39−41^ methods. Details are given below. These two methods were chosen as appropriate multi- and single-reference levels of theory based on our previous work on the vertical excitation energies of *o*NBA.^33^ We employ ADC(2) for its attractive computational cost and its ability to describe the bright ππ* states in the Franck–Condon region accurately. MS-CASPT2 has a much higher computational demand—especially if the bright ππ* states are targeted^33^—but allows the investigations of the bond reformation steps during ketene formation, which is not doable with ADC(2). The nonadiabatic dynamics simulations in the second step were only performed with ADC(2), due to the high cost and unavailability of gradients of MS-CASPT2. All ADC(2) calculations were performed with TURBOMOLE 7.0,^42^ all MS-CASPT2 calculations with OpenMolcas 18.0.^43^

### Electronic Structure Level of Theory

**Figure 2:**
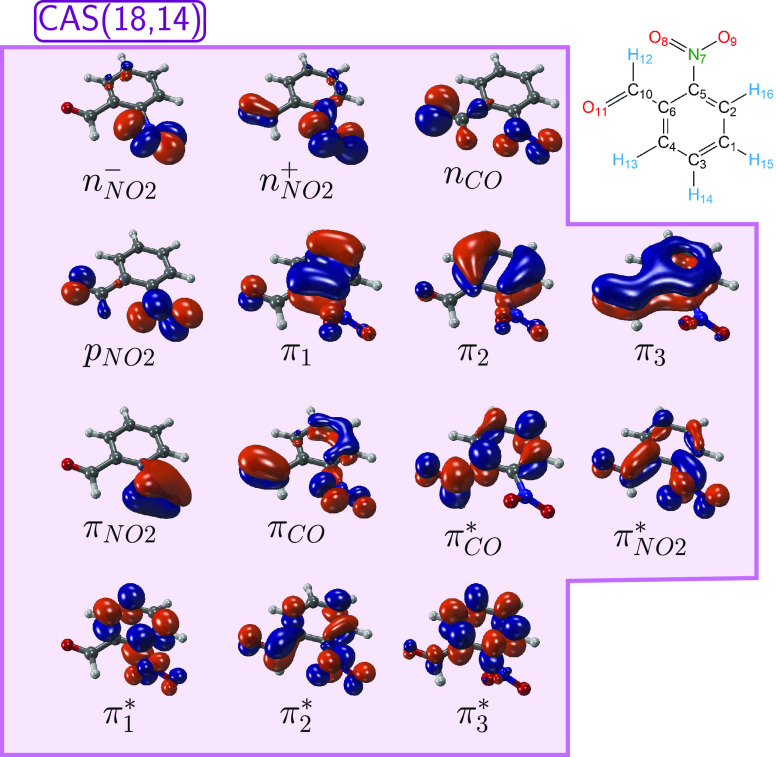
Active space employed for the vertical excitation calculations. The top right image shows the atom numbering used below. This active space was already discussed in ref (33).

The MS-CASPT2 calculations used in the PES exploration employ the cc-pVTZ basis set^44^ and a complete active space (CAS) with 18 electrons in 14 orbitals (CAS(18,14)), which is the same as used in our previous study.^33^ The active orbitals are presented in Figure 2. They include six π/π* orbitals of the aromatic ring, three π/π* orbitals of the NO_2_ group, two lone pair orbitals of the NO_2_ group, two π/π* orbitals of the carbonyl group, and one lone pair orbital of the carbonyl group. All state-averaging and multistate treatments in our calculations were carried out with either 12 singlet^33^ or 12 triplet states, denoted as MS(12,12)-CASPT2. The Cholesky decomposition technique^43,45^ was employed to speed up the calculations. The imaginary level shift^46^ was set to 0.2 a.u., and the ionization potential-electron affinity (IPEA) shift^47^ was set to 0.25 a.u., as is recommended for triple-zeta basis sets.^48^ Further discussion on the MS-CASPT2 level of theory and on its accuracy and robustness in treating the low-lying excited states of *o*NBA can be found in ref (33).

The ADC(2) calculations used for PES exploration and nonadiabatic dynamics simulations employ the smaller cc-pVDZ basis set. The default auxiliary basis set assigned by Turbomole was used for the resolution-of-identity technique.

### Potential Energy Surface Exploration

Table 1 gives an overview of all critical points considered in this work. Because the excited state PESs are quite closely spaced, we only optimized minima on the S_0_, S_1_, and T_1_ adiabatic PESs. Optimization attempts for an S_2_ or S_3_ minimum failed. Based on the identified bright singlet state (S_4_ for ADC(2)), MECIs for the full relaxation cascade S_4_ → S_1_ were optimized. For ISC, we optimized two S_1_/T_2_ MECPs and a T_2_/T_1_ MECI. Finally, to investigate the relaxation and ketene formation processes, we optimized three S_1_/S_0_ MECIs and three analogous T_1_/S_0_ MECPs.

**Table 1:** Overview of Considered Critical Points of *o*NBA and Their Numerical Labels Used Below

label	geom.	label	geom.
1	S_0_min	12	S_1_/T_2_^P^
2	S_5_/S_4_	13	S_1_/T_2_
3	S_4_/S_3_	14	T_2_/T_1_
4	S_3_/S_2_	15	T_1_min
5	S_2_/S_1_^P^	16	T_1_/S_0_^CO^
6	S_2_/S_1_	17	T_1_/S_0_^NO^
7	S_1_min	18	T_1_/S_0_^HT^
8	S_1_/S_0_^CO^	19	T_1_^bir^min
9	S_1_/S_0_^NO^		
10	S_1_/S_0_^HT^		
11	S_0_^bir^min		

The excited state minima, MECIs, and MECPs were optimized through the SHARC package,^34−36^ using the external optimizer feature of ORCA 4.2.1,^49^ which was provided with gradients from ADC(2), CASSCF, or MS-CASPT2 through SHARC. CASSCF was only used for preoptimizations. Gradients for MECI optimizations were computed with a penalty-function approach^50^ and for MECP calculations with the Bearpark–Robb–Schlegel algorithm.^51^ All optimizations used the smaller cc-pVDZ basis set (see Tables S1 and S2 in the Supporting Information (SI) for a comparison with the larger cc-pVTZ basis). We attempted to optimize most of the structures with both MS-CASPT2 and ADC(2), with few exceptions, as detailed in Table S3 in the SI. Note that the MS-CASPT2 optimizations were generally done with two smaller ASs—a CAS(12,9) with five nitro group orbitals and four π system orbitals and a CAS(12,10) with two nitro group orbitals, four π system orbitals, three aldehyde group orbitals, and the aldehyde σ_CH_ orbital.

The optimized geometries were connected through linear interpolation of internal coordinates (LIIC), using Gaussian Z-matrix coordinates (via OpenBabel^52^) to construct relevant PES segments. These were used to check for AS consistency and to estimate activation barriers, where the barriers found in the LIIC scans are upper limits to the true barriers. All PES segments were recomputed with ADC(2)/cc-pVDZ and MS(12,12)-CASPT2(18,14)/cc-pVTZ (imaginary shift increased to 0.2 a.u.) to obtain consistent energy profiles of the proposed reaction pathways.

### Nonadiabatic Dynamics Simulations

Nonadiabatic dynamics simulations including IC and ISC were carried out with the SHARC package.^34−36^ 1000 initial conditions were sampled from the ground state Wigner distribution^53,54^ computed from an MP2/cc-pVDZ frequency calculation. At each initial geometry, an ADC(2)/cc-pVDZ vertical excitation calculation of 12 excited singlet and 12 triplet states was performed to generate an ensemble-broadened absorption and density-of-state spectra. The initially excited singlet state for each initial condition was selected stochastically^55^ from the energy window of 4.5–4.7 eV (264–276 nm), which is centered on the second absorption band in analogy to previous transient absorption experiments.^19^ 96 trajectories—86 (90%) of them starting in S_4_, 9 (9%) in S_3_, and 1 (1%) in S_2_—were propagated for 300 fs at ADC(2)/cc-pVDZ level of theory including six singlet (five + ground state) and eight triplet states (chosen based on the density-of-state spectrum). This smaller set of initial conditions still samples the ground state minimum well, as shown in Figure S1. The nuclear dynamics time step was 0.5 fs and 25 substeps were employed for the integration of the electronic equation of motion (0.02 fs) with the local diabatization method,^56^ using wave function overlaps computed with the WFoverlap program.^57^ Spin-orbit couplings (SOCs) were computed as previously reported.^58^ To conserve the total energy, the entire velocity vector was rescaled. An energy-based decoherence correction was applied.^59^

Trajectories were analyzed in terms of electronic spin-free populations,^35^ CT character using TheoDORE,^60,61^ and relevant geometric parameters. The CT numbers were computed for three fragments (C_6_H_4_, NO_2_, CHO) for each trajectory, combined with the electronic coefficients, and finally averaged over all trajectories. Furthermore, the energies and oscillator strengths (relative to S_0_) from all trajectories and time steps were used to compute a time-dependent luminescence spectrum via Gaussian convolution (full width at half-maximum of 0.25 eV).

## Results and Discussion

### Vertical Excitations

The vertical excitation energies and characters of the electronic states of *o*NBA are described in detail in ref (33). Here, we only briefly recapitulate the most relevant information needed for the subsequent discussion. Table 2 shows the vertical excitation results with ADC(2) and MS-CASPT2(18,14); Table S4 in the SI provides some additional wave function descriptors. Figures S2 and S3 show the transition densities. Table 2 also compares the experimental absorption spectrum, which contains four progressively more intense absorption bands/shoulders at about 3.7 eV (least intense), 4.4 eV, 5.2 eV, and 5.7 eV (most intense).^24^

**Table 2:** Vertical Excitation Energies (Δ*E*, in eV), Oscillator Strengths (*f*_osc_), and Leading Characters of the Different States of *o*NBA^33^^a^

ADC(2)/cc-pVTZ				MS(12,12)-CASPT2(18,14)/cc-pVTZ				bexp.^24^
state	Δ*E*	*f*_osc_	character^c^	state	Δ*E*	*f*_osc_	character^d^	Δ*E*
S_1_	3.48	0.001	*n*_CO_π*	S_1_	3.58	0.001	*n*_CO_π*	
S_2_	3.63	0.008	*n*_NO_2__^–^π*	S_2_	3.77	0.004	*n*_NO_2__^–^π*	3.7
S_3_	4.17	0.001	*n*_NO_2__^+^π*	S_3_	4.20	0.001	*n*_NO_2__^+^π*	
S_4_	4.66	0.024	ππ* (*L*_*b*_)	S_4_	4.54	0.003	ππ* (*L*_*b*_)	4.4
S_5_	5.14	0.006	*n*_CO_π* (CT)	S_6_	6.10	0.001	*n*_CO_π* (CT)	
S_6_	5.42	0.149	ππ* (*L*_*a*_)	S_5_	5.58	0.058	ππ* (*L*_*a*_)	5.2
T_1_	3.14		*n*_CO_π*	T_1_	3.37		*n*_CO_π*	
T_2_	3.31		*n*_NO_2__^–^π*/ππ* (*L*_*a*_)	T_2_	3.49		ππ*(NO_2_)	
T_3_	3.67		ππ* (NO_2_)	T_3_	3.53		*n*_NO_2__^–^π*/ππ* (*L*_*a*_)	
T_4_	3.87		*n*_NO_2__^+^π*/ππ* (*L*_*a*_)	T_4_	3.70		*n*_NO_2__^+^π*/ππ* (*L*_*a*_)	

a More details on state characters and weights can be found in Table S4 and Figures S2 and S3 in the SI.

b Experimental results from Gaussian fits to the absorption spectrum.^24^

c See transition densities in Figure S2 in the SI.

d See transition densities in Figure S3 in the SI.

The three lowest excited singlet states all have local *n*π* character. At the Franck–Condon geometry the S_1_ is an excitation of the carbonyl lone pair (*n*_CO_π*), whereas S_2_ and S_3_ are linear combinations of excitations from the two lone pairs of the nitro group (*n*_NO_2__^–^π* and *n*_NO_2__^+^π*). Due to the nonplanarity of *o*NBA in the ground state, these states acquire small ππ* contributions and some nonzero oscillator strengths, giving rise to the weak experimental absorption at 3.7 eV. The description of these states is robust regarding the details of the electronic structure calculation,^33^ considerably simplifying the elucidation of the ketene formation mechanism.

The S_4_ state is the lowest predominantly ππ* state (the *L*_*b*_ state in Platt’s nomenclature^62^) of *o*NBA at an energy of about 4.5 eV (assigned to the absorption band at 4.4 eV). The S_5_ and S_6_ states are a charge-transfer *n*_CO_π* and the second ππ* state (*L*_*a*_), with different ordering between ADC(2) and MS-CASPT2, but a decent agreement of the ππ* energy with experiment (5.2 eV). The inconsistent description of the charge-transfer *n*_CO_π* between the two methods was already observed in our previous study,^33^ where we argued that ADC(2) fails in describing this dark state due to its substantial double excitation contributions. We estimate that the charge-transfer *n*_CO_π* is actually located at 6–7 eV,^33^ too high to affect the processes studied in the present work. While the two methods qualitatively agree on the state characters and energies, they predict rather different oscillator strengths—with ADC(2) predicting the absorption spectrum much better.^33^ The reason is that the SA(12)-CASSCF results underlying the MS-CASPT2 calculation are missing several bright ππ* states, which diminishes the oscillator strengths of the MS-CASPT2 solutions. Recovering the oscillator strengths would require performing XMS-CASPT2 on the order of 40 states, which is computationally too demanding for the present study. Thus, for the description of the decay from the bright ππ* states to the low-lying states we rely predominantly on ADC(2). The investigation of the ketene formation within the low-lying states is not affected by the shortcomings of MS(12)-CASPT2 to describe the high-lying ππ* states.

The lowest triplet states are found to be of *n*_CO_π*, ππ* (NO_2_), *n*_NO_2__π*, and ππ* (*L*_*a*_) characters. A particularly interesting state here is the local ππ* (NO_2_) character, whose singlet state has a very high energy above 6 eV,^33^ while the triplet state has low energy and plays an important role in the ISC dynamics (see below). The large difference in energy between the singlet and triplet states can be explained by the self-repulsion of the transition density, which is zero for the triplet state.^63^ Overall, the two methods predict comparable triplet states, albeit with slightly different ordering due to the small energy differences of the T_2_ and T_3_. Nonetheless, both methods provide energies in good agreement with each other, so we are confident that the accuracy of the triplet states is sufficient to study ISC, relaxation, and ketene formation.

### Optimized Excited State Minima and Crossing Points

**Figure 3:**
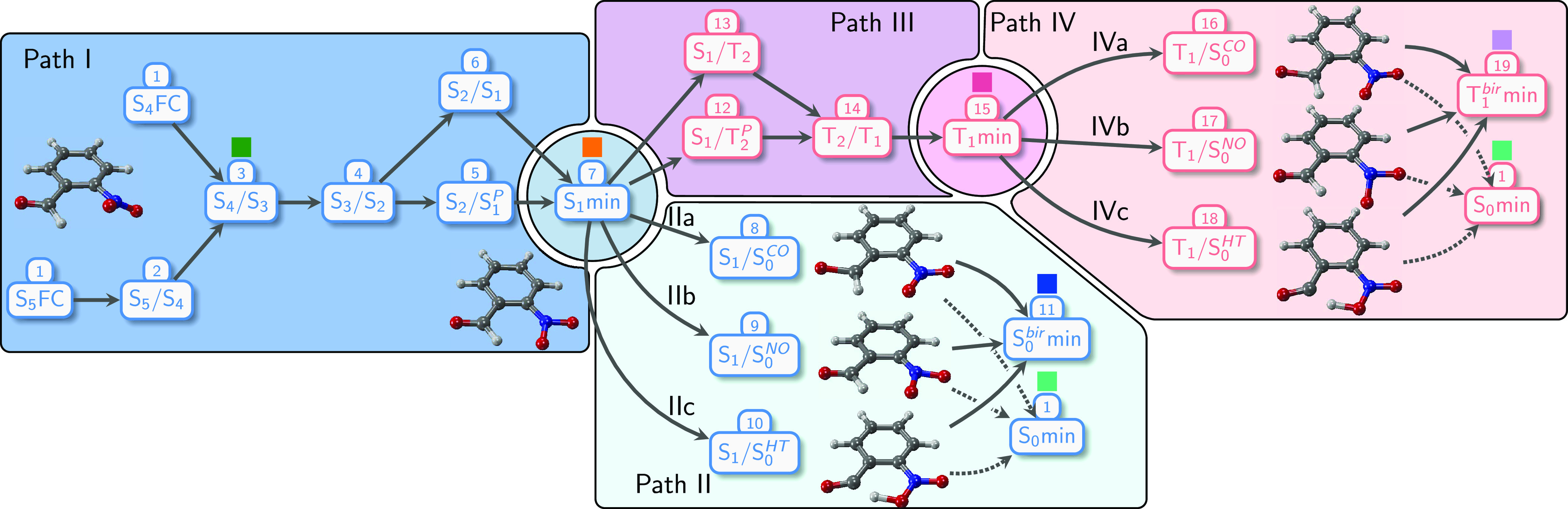
Schematic roadmap that provides an overview of the critical geometries optimized with ADC(2) and MS-CASPT2, as well as paths between them. Colored squares denote some of the optimized geometries that appear in more than one PES scan in Figure 4.

Figure 3 provides a roadmap of the 19 critical geometries optimized in the present work. For ease of reference, the schematic is divided into four stages (called “Paths”). Path I represents the near-barrierless relaxation cascade from the initially populated bright states. For ADC(2), we only considered S_4_ as the initial state, whereas for MS-CASPT2, we considered both S_4_ and S_5_ as potential initial states. Path II subsumes all deactivation paths of the S_1_ minimum through several MECIs, leading either back to the initial *o*NBA geometry or to the biradical intermediate. Alternatively, Path III describes ISC from S_1_, followed by triplet IC, and ends at the T_1_ minimum. Finally, Path IV collects the different deactivation channels from the T_1_ minimum, which to some extent mirror the channels in Path II. Energies at all minima and geometric parameters for all structures obtained with ADC(2) and MS-CASPT2 are collected in Tables S5 to S8 in the SI. Corresponding transition densities are given in Figures S4 and S5.

The ground state geometry (optimized with MP2/cc-pVDZ) agrees with previous X-ray^64^ and computational^12,22,23,25^ findings, exhibiting nonzero dihedral angles of the aldehyde (∼8°) and nitro groups (∼35°) with the ring due to steric effects. The nonplanarity induces mixing between *n* and π orbitals, contributing to the mixed electronic structure encountered for the vertically excited states discussed previously.^33^

The obtained crossing points in Path I—S_5_/S_4_ (MS-CASPT2 only), S_4_/S_3_, S_3_/S_2_, and two S_2_/S_1_ crossings (all optimized with ADC(2))—show relatively little variation in their geometries. The crossing between ππ* (*L*_*b*_) and *n*_NO_2__^+^π* states (S_4_/S_3_) exhibits nitro and aldehyde groups in the molecular plane. The lower crossing points—between *n*_NO_2__^+^π*, *n*_NO_2__^–^π*, and *n*_CO_π* (S_3_/S_2_ and both S_2_/S_1_)—are characterized by a planar ring and a nitro group rotated out of the molecular plane. The two S_2_/S_1_ crossings differ in their geometry and wave function characters—at the S_2_/S_1_^P^ (P for planar) with a planar (i.e., nonpyramidalized) nitro group the *n*_CO_π* and *n*_NO_2__^–^π* states cross. The other S_2_/S_1_ crossing (without superscript) exhibits a pyramidalized nitro group but is energetically less favorable. No true minima on the adiabatic PESs of the S_2_ to S_5_ states could be located—in contrast to ref (12) who reported a ππ* minimum on the S_3_ PES—as all optimization attempts ended at crossing points. Path I ends at the S_1_ minimum (*n*_NO_2__^–^π*), which is fully planar, with the aldehyde hydrogen atom (H_12_) in the same plane as the nitro oxygen atom (O_8_), prearranging *o*NBA for the HT reaction.^12^

Within Path II, we have identified three relevant S_1_/S_0_ MECIs. The first intersection, S_1_/S_0_^CO^ (Path IIa), is planar and exhibits an elongated C_10_–O_11_ bond but no sign of HT. It is a crossing point of the *n*_CO_π* state with the closed-shell ground state. While the ADC(2) geometry has a rather low energy, the MS-CASPT2 optimized geometry is energetically inaccessible with a barrier of almost 2 eV (Table S7), supposedly due to a very short O_11_–H_13_ distance (distance between carbonyl O and aromatic H). The S_1_/S_0_^NO^ crossing (Path IIb)—involving the *n*_NO_2__^–^π* and closed-shell states—is also planar and shows no sign of HT, but is distinguished by an acute O_8_–N_7_–O_9_ bond angle of about 90° and a shortened N_7_–C_5_ bond. This crossing is reminiscent of a S_1_/S_0_ CI of nitrobenzene,^65^ which has been predicted to lead to recovery of the ground state nitro species. However, for *o*NBA the S_1_/S_0_^NO^ crossing was not reported previously to the best of our knowledge. The third crossing is termed S_1_/S_0_^HT^ and corresponds to the HT pathway first reported by Migani et al.^12^ in 2011. The geometry is planar, with an elongated C_10_–H_12_ bond and a newly formed O_8_–H_12_ bond (see Tables S6 and S8in the SI). The optimized geometries show good agreement between the two levels of theory. At this crossing, the transition density shows excitations between the *n*_CO_, σ_CH_ (aldehyde), and π_NO_2__^*^ orbitals. From each of the three S_1_/S_0_ MECIs, *o*NBA could potentially return back to the S_0_ minimum or (with different propensities) continue toward the biradical structure in the S_0_, as discussed below.

As indicated in Path III in Figure 3, from the S_1_ minimum, there are also two potential pathways for ISC, given by the two MECPs S_1_/T_2_^P^ (“planar”) and S_1_/T_2_. Both geometries feature a nitro group that is rotated out of the molecular plane (by about 19 °); however, in the latter point, the nitro group is also pyramidalized. In that sense, they resemble the S_1_/S_2_^P^ and S_1_/S_2_ MECIs. The S_1_/T_2_^P^ MECP enables the transition between the ^1^*n*_NO_2__^–^π* and ^3^*n*_CO_π* characters, with negligible SOCs (7 cm^–1^ at ADC(2) level). This point could not be located with MS-CASPT2. The S_1_/T_2_ MECP couples the ^1^*n*_NO_2__^–^π* and ^3^π_NO_2__π* characters via sizable SOCs of 50 or 70 cm^–1^ (ADC(2) or MS-CASPT2), as shown in Tables S9 and S10 in the SI. We also tried optimizing various other singlet–triplet crossings for higher states (S_2_ with T_3_ and T_4_, S_3_ with T_5_ and T_6_, S_4_ with T_7_), but we did not obtain any with relevant SOCs. Hence, we assume that ISC would predominantly occur from the S_1_ minimum via the S_1_/T_2_ MECP. Once in the T_2_ state, IC via a T_2_/T_1_ crossing leads efficiently to the T_1_ minimum (^3^*n*_NO_2__^–^π* character). This minimum somewhat resembles the S_1_ minimum but is not fully planar at ADC(2) level (the nitro group is rotated and slightly pyramidalized) and thus might be less efficient for HT.

From the T_1_ minimum (Path IV), we identified three T_1_/S_0_ MECPs that each resembles one of the three S_1_/S_0_ MECIs of Path II. T_1_/S_0_^CO^ features a strong C_10_–O_11_ bond elongation and a very high MS-CASPT2 energy. T_1_/S_0_^NO^ displays a very acute O_8_–N_7_–O_9_ bond angle, and additional pyramidalization of the nitro group (unlike the corresponding S_1_/S_0_ crossing). T_1_/S_0_^HT^ is planar, with the aldehyde H half-way between the aldehyde C and the nitro O atoms. Hence, the T_1_/S_0_^HT^ crossing might enable HT with a certain probability after ISC to the S_0_ occurred. Alternatively, the molecule could stay in the triplet during HT.

Additionally, we investigated the possibility of HT occurring either in the singlet (Path II) or the triplet manifold (Path IV), guided by previous theoretical investigations.^12,65^ This channel was only studied with MS-CASPT2, as the ground state acquires a strong biradical character during the aldehyde-ketene transformation.^12^ We optimized two structures, S_0_^bir^ min and T_1_^bir^ min, which are the singlet and triplet versions of the “Bir” structure of ref (12). Both geometries exhibit elongated C_10_–H_12_ bonds and a large *a*_11,10,6_ bond angle (C–C–O), which is characteristic of the ketene formation. As S_0_^bir^ min is located on the S_0_ adiabatic surface, it is photochemically accessed from an S_1_/S_0_ CI, and we investigated all three intersections. The T_1_^bir^ min is formally accessible from the T_1_ min via a transition state. However, instead of optimizing such a transition state, we assumed that the T_1_ min–T_1_^bir^ min conversion pathway passes close to a T_1_/S_0_ crossing.

### Excited State Potential Energy Surfaces

**Figure 4:**
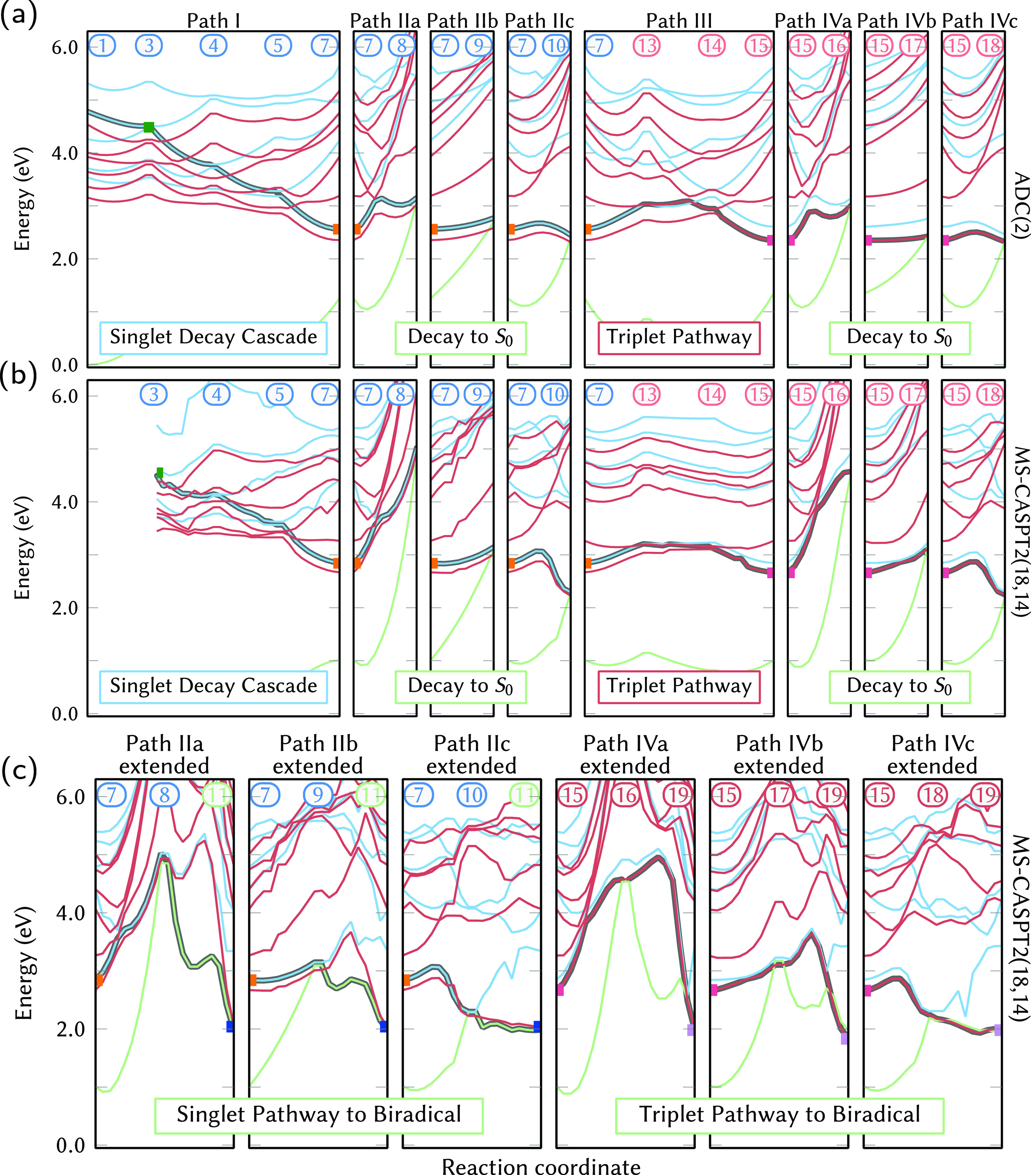
Relaxation pathways obtained at ADC(2)/cc-pVDZ (a) and MS(12,12)-CASPT2(18,14)/cc-pVTZ (b, c) levels of theory. Green indicates the S_0_, blue excited singlet states, and red triplet states. The numeric labels indicate the different geometries along the scan, as given in Figure 3 (and Table 1). The reaction coordinate is given by LIIC scan segments between the geometries indicated by the numerical labels. Colored squares indicate geometries that are shown in multiple panels. Note that the ADC(2) and MS-CASPT2 scans are based on different geometries, as given in Table S3.

Figure 4 presents LIIC scans of all pathways presented in Figure 3. The scans were computed side-by-side with ADC(2) and MS-CASPT2(18,14). The main goals of this comparison are (i) scrutinizing the relative importance of the different relaxation pathways and (ii) mutually validating the two methods and scrutinizing their robustness. We expect that ADC(2) is reliable for Paths I and III, but not for Paths II and IV due to the presence of near-degeneracies of S_0_ with the excited states. In turn, Path I might not be reliably described with MS-CASPT2 due to the difficulties in describing the higher-lying states;^33^ MS-CASPT2 is expected to describe Paths II, III, and IV appropriately.

#### ADC(2)

The relaxation pathway from the initially excited S_4_ (ππ*) state to the S_1_ minimum (Path I) is shown in Figure 4a (leftmost segment). Since this path is barrierless and no true minima in S_2_, S_3_, or S_4_ were found, we expect very rapid decay to the S_1_ minimum. This is consistent with findings from femtosecond fluorescence spectroscopy,^21,27^ where the first time constant (τ_1_ < 100 fs) was assigned to the depletion of the bright ππ* state. The transition from S_2_ to S_1_ can occur via two different MECIs, S_2_/S_1_^P^ (geometry 5) or S_2_/S_1_ (geometry 6), which are compared in Figure S6a,b. Based on the slope of the PESs and the required nuclear motion, we estimate that S_2_/S_1_^P^ dominates decay to S_1_, even though it involves one additional change of character (*n*_NO_2__^+^π* → *n*_CO_π* → *n*_NO_2__^–^π*).

As depicted in Figure 4a, Path I exhibits a number of singlet–triplet crossings (S_3_/T_6_, S_3_/T_5_, S_2_/T_4_, S_2_/T_3_), which potentially could enable early ISC before reaching the S_1_. Examination of these crossing points revealed only small SOCs (<15 cm^–1^). Also considering the absence of any true minima in S_2_, S_3_, or S_4_, based on the scans we expect that IC to S_1_ is predominant over ISC from the higher singlet states.

Path II entails three PES segments (Figure 4a, Paths IIa, IIb, IIc) to reach the respective S_1_/S_0_ MECI, from where the system could return to the *o*NBA ground state or continue to the ketene form. These segments involve energy barriers of 0.51 eV (S_1_/S_0_^CO^), 0.18 eV (S_1_/S_0_^NO^), and ≤0.11 eV (S_1_/S_0_^HT^). However, as the MECIs are not properly described with ADC(2), we discuss these crossings in the MS-CASPT2 Section.

Alternatively, from the S_1_ minimum two S_1_/T_2_ MECPs can be reached. One of these two pathways is shown in Figure 4, whereas both are compared in Figure S6c,d. The pathway via the S_1_/T_2_ shown in Figure 4 involves the larger barrier from the S_1_ minimum (0.47 eV) but also larger SOCs (50 cm^–1^), leading to a higher probability to switch from the singlet to the triplet manifold. The alternative pathway via S_1_/T_2_^P^ involves a smaller barrier (0.39 eV) but much smaller SOCs (7 cm^–1^). These findings indicate that ISC from the S_1_ minimum is probably not very efficient and will likely be inferior to IC to the S_0_, at least in the gas phase. However, based on our results, S_1_ → T_2_ ISC is the most likely explanation for the experimentally observed triplet yield.^27^ Once in the triplet manifold, the T_1_ minimum can be reached barrierlessly from T_2_.

As the S_1_ and T_1_ PESs are rather similar, Path IV is a close analogue of Path II. The energy barriers from the T_1_ minimum to the T_1_/S_0_ MECPs are 0.64 eV (T_1_/S_0_^CO^), 0.08 eV (T_1_/S_0_^NO^), and < 0.15 eV (T_1_/S_0_^HT^) at ADC(2) level. As there are no SOCs for the S_0_ available in Turbomole, the importance of these crossings is discussed below with the MS-CASPT2 results.

#### MS-CASPT2

The MS-CASPT2(18,14) scans of Path I are shown in Figure 4b. Unfortunately, due to the usage of a smaller active space in the optimization, no accurate S_5_/S_4_ MECI could be optimized (energy gap of 0.3 eV). Thus, the additional PES segment from the S_5_ state (initially excited state with MS-CASPT2) to the S_4_/S_3_ crossing is shown in Figure S6e. In any case, the experimental observation of a fast ππ* decay (τ_1_ < 100 fs)^21,27,28^ indicates that decay from the bright state should be easily possible. Starting in the S_4_, MS-CASPT2 predicts an essentially barrierless pathway to the S_1_ minimum, just like ADC(2).

One of the main motivations for employing MS-CASPT2(18,14) was to ensure a proper description of the S_1_/S_0_ and T_1_/S_0_ crossing points (Paths II and IV) that are critical for the description of *o*NBA’s photochemistry. MS-CASPT2 shows that the S_1_/S_0_^CO^ deactivation channel (Path IIa) that was predicted by ADC(2) is actually inoperative in *o*NBA, due to an energy barrier of more than 2 eV. Similar results for other carbonyls have been reported previously in the literature.^66^ The S_1_/S_0_^NO^ (IIb) and S_1_/S_0_^HT^ (IIc) crossing points exhibit much smaller barriers of 0.28 and ≤0.22 eV, respectively, at MS-CASPT2 level—consistent with ADC(2). As previously reported for nitrobenzene,^65^ the S_1_/S_0_^NO^ CI leads to the regeneration of the initial nitro species because of the “sloped” topography of the intersection.^67^ On the contrary, the S_1_/S_0_^HT^ CI is “peaked” (the S_0_ and S_1_ surfaces are sloped in different directions in Figure 4b, IIc) and therefore enables photoproduct formation. The barrier heights for S_1_/S_0_^NO^ and S_1_/S_0_^HT^ are very similar, which could be a reason for the approximately 50% quantum yield of ketene formation after photoexcitation.^11,13^

The barrier for ISC in Path III is found to be about 0.37 eV at MS-CASPT2 level, with an impressive SOC of 70 cm^–1^, consistent with ADC(2). Considering that the ISC activation barrier is larger than the one for IC to the S_0_, it is easily understandable that ISC is only a minor process in *o*NBA. The further IC within the triplet states is barrierless with MS-CASPT2, just like at ADC(2) level.

The last segments in Figure 4b show Path IV, the pathways from the T_1_ minimum to the T_1_/S_0_ MECIs. As with ADC(2), at the MS-CASPT2 level Path IV is very similar to Path II. The T_1_/S_0_^CO^ is shown to be energetically unreachable (1.6 eV above T_1_ minimum), whereas T_1_/S_0_^NO^ and T_1_/S_0_^HT^ are both accessible (barriers of 0.44 and 0.20 eV, respectively). The SOCs at the two latter MECPs are very large (64 cm^–1^ for T_1_/S_0_^NO^, 35 cm^–1^ for T_1_/S_0_^HT^), which indicates that reverse ISC might remove a small fraction of the triplet population before it can react toward the biradical structure.

The extended Paths IIa–c in Figure 4c (left) show how the singlet biradical structure could be formed from the S_1_/S_0_ MECIs. Here, both Path IIb and IIc in principle allow the formation of this biradical structure; however, Path IIb involves a second barrier and thus is more likely to lead back to the S_0_ minimum of *o*NBA. In Figure 4c (right), a similar picture is given for the triplet Paths IVa–c. Due to a rather high barrier in the T_1_ in Path IVb, in the triplet, only Path IVc seems viable. Along this path, the triplet biradical structure can form quickly from the ^3^*n*_NO_2__^–^π* minimum after crossing a small barrier and without any nonadiabatic transition, unlike in the singlet pathway. Assuming that the molecule only stays for short times close to the T_1_/S_0_ MECPs, we expect that most triplet population eventually ends up in the biradical T_1_^bir^ minimum. This latter minimum can be identified as the basin where the triplet population is trapped for 220 ps^27^ before forming the ketene, due to the slow reverse ISC from the triplet to the closed-shell singlet ketene ground state. A similar mechanism was already discussed previously^68^ for ortho-nitrobenzylacetate.

### Nonadiabatic Dynamics Simulations

In this section, we present dynamics simulations using the SHARC method^34−36^ at the ADC(2)/cc-pVDZ level of theory, primarily to describe the IC processes from the initially excited state to the S_1_ minimum and any concurrent ISC processes (Paths I and III). As discussed above, the single-reference method ADC(2) cannot accurately describe the pathways where the S_0_ becomes degenerate with the excited states, so Paths II and IV are not the focus of these simulations. We discuss below in a separate section the trajectories that attempted to follow these paths.

#### Simulated Absorption Spectrum

**Figure 5:**
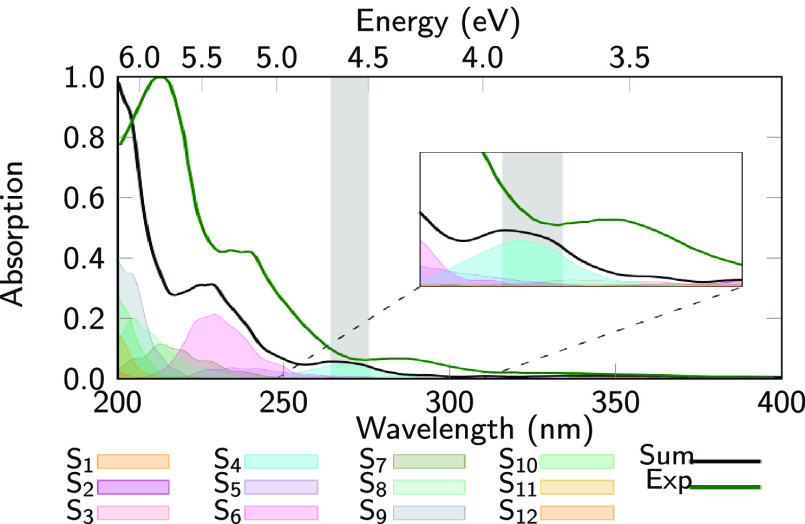
Computed absorption spectrum (black) of *o*NBA at ADC(2)/cc-pVDZ level of theory, based on 1000 initial conditions sampled from a Wigner distribution. Individual state contributions are given as shaded areas. The gray box denotes the excitation window (4.5–4.7 eV), primarily exciting the S_4_ state (ππ* *L*_*b*_). The dark-green line shows the experimental absorption spectrum.^24^

The simulated absorption spectrum of *o*NBA at ADC(2) level is shown in Figure 5 (black line). Apart from a systematic energy shift of 0.3 eV, the spectrum exhibits excellent agreement with the experimental one (green), reproducing the energy gaps, relative intensities, and bandwidths of the four absorption bands (at 340, 290, 240, and 210 nm).^24^ As already discussed previously,^33^ this shows that ADC(2) provides a good description of the excited singlet states of *o*NBA in the Franck–Condon region.

For the nonadiabatic dynamics simulations, we excite *o*NBA in the second absorption band, corresponding to the excitation of the lowest ππ* state,^33^ respectively, to the 260–270 nm (4.7 eV) excitation wavelength used in previous experiments.^18−21,27^ To obtain a sufficient number of trajectories, we set the excitation window to 4.5 eV (276 nm) to 4.7 eV (264 nm). In this window, the brightest state is the one with ππ* (*L*_*b*_) character, which at most initial condition geometries is the S_4_ adiabatic state. From the 1000 initial conditions, 96 were selected^55^ (86 starting in S_4_, 9 in S_3_, 1 in S_2_).

#### Electronic Evolution

The 96 selected initial conditions were propagated with SHARC for 300 fs. 67 trajectories successfully ended at 300 fs, whereas 29 trajectories terminated earlier. The majority of crashes were due to convergence problems close to near-degeneracies with the S_0_ (see Path II in Figure 4a). We will first discuss the initial decay and ISC as represented by the entire swarm of trajectories. Subsequently, the crashed trajectories will be discussed separately with respect to potential relaxation channels to S_0_.

**Figure 6:**
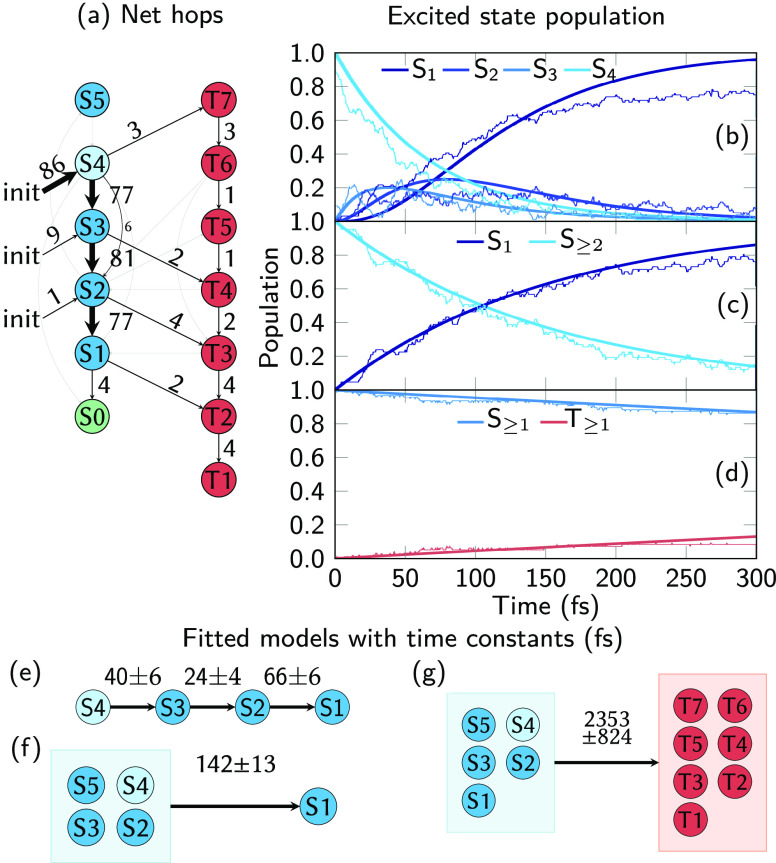
(a) Net population transfers extracted from the dynamics simulation. (b–d) Electronic populations (thin lines) fitted with three different unimolecular first-order kinetic models. (e–g) Representations of the three kinetic models and fitted time constants (in fs, with uncertainty from bootstrapping^69^). Singlets are given in shades of blue and triplets in shades of red.

In Figure 6, we show the evolution of the adiabatic electronic populations based on all 96 trajectories (including the crashed ones). The net number of hops between the different adiabatic states (panel (a)) clearly show that the majority (≥80%) of the trajectories is initially excited to S_4_ and sequentially decays through S_3_ and S_2_ to S_1_. This confirms the predominant initial relaxation pathway of *o*NBA to be S_4_ → S_3_ → S_2_ → S_1_ (in terms of adiabatic states). Additionally, it can be seen that four trajectories returned to the ground state, although the S_1_–S_0_ coupling is not correctly described at ADC(2) level (these four trajectories crash soon after switching to S_0_ due to convergence issues).

Overall, only seven of 96 trajectories (7%) underwent ISC to the triplet states within 300 fs, at various different singlet–triplet crossings: S_4_/T_7_, S_3_/T_4_, S_2_/T_3_, and S_1_/T_2_. All of these crossing points were found in the course of the potential energy surface exploration as well; however, the PES scans (Figure 4) suggested that ISC from higher singlets is probably less relevant than ISC from S_1_. The dynamics results instead provide evidence that ISC can occur with small probability during the entire initial relaxation cascade, although no dominant MECP/ISC channel can be identified due to the small number of singlet–triplet-hops. In any case, trajectories undergoing ISC eventually end up in T_1_ due to a deactivation cascade similar to the one shown in Path I in Figure 4a.

In order to estimate a confidence interval for the ISC yield of our calculations, we employ the Wilson score interval.^70^ The interval is given by , where *n* = 96 is the number of trajectories, *n*_ISC_ = 7 is the number of trajectories undergoing ISC, and λ = 1.96 is a parameter that corresponds to a 95 % confidence level. We thus estimate the ISC yield as 9 ± 5%. This should be regarded as a lower bound, as more trajectories might undergo ISC later than 300 fs after excitation. This statistical analysis shows that even with our relatively small number of trajectories undergoing ISC, we can say with high confidence that ISC in *o*NBA does occur. By applying the equation separately to ISC from higher states (5 trajectories, 7 ± 5 %) and from S_1_ (2 trajectories, 3 ± 3 %), we find that our simulations do not allow estimating whether ISC from higher states or from S_1_ will dominate.

Next, we extracted time constants for the population transfer in *o*NBA from the adiabatic populations, as shown in Figure 6b,d. For each of the three panels, we performed a global population fit using the first-order sequential kinetic models shown in panels (e–g). The first model (panels (b) and (e)) allows extracting time constants for the relaxation cascade from adiabatic S_4_ to S_1_; these time constants are about 40 fs (S_4_ → S_3_), 25 fs (S_3_ → S_2_), and 65 fs (S_2_ → S_1_). The first of these time constants is an approximation to the decay time of the initially populated bright ππ* state.

In the second kinetic model (Figure 6c,f), we obtain the overall decay time constant of the higher singlet states—which is equivalent to the rise time of S_1_—as about 140 fs. We are not aware of an experimental time constant that directly probes the appearance of the lowest *n*π* state. The time constant for ketene formation is reported to be a few 100 fs^20^ (τ_2_, typically about 400 fs^18,19,21^). This is consistent with a mechanism where first the *n*π* minimum is reached in 140 fs and subsequently the ketene is accessed via the small barrier in Path IIc (see Figure 4c).

The third kinetic model (Figure 6d,g) provides an estimate for the overall ISC time scale in *o*NBA. Due to the small number of trajectories undergoing ISC and the short simulation time, the ISC time constant of 2.4 ps has a rather large uncertainty of 0.8 ps. Nonetheless, the simulations provide strong evidence that the IC cascade from S_4_ to S_1_ is significantly faster than ISC. The obtained few-ps time constant is also consistent with results reported for other nitro-aromatic compounds^71^ and nicely matches an experimental estimate of the ISC time constant in *o*NBA (2.7 ps).^27^

**Figure 7:**
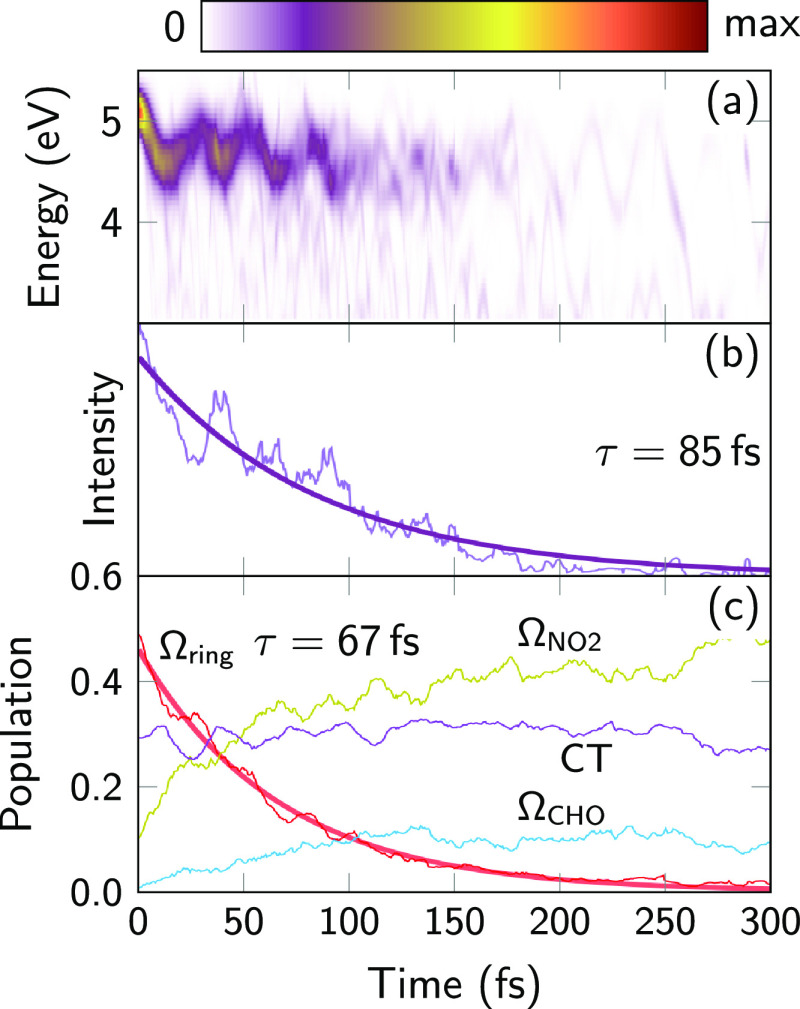
(a) Simulated fluorescence spectrum based on energy differences and oscillator strengths between active state and ground state. The data was convoluted with Gaussians of 0.25 eV full width at half-maximum, but not convoluted along the time axis. (b) Integrated fluorescence signal between 3.4 and 5.5 eV and monoexponential fit with τ = 85 fs. (c) Time evolution of the CT character of the electronic wave function in terms of local excitations on the ring, NO_2_, CHO, and CT excitations. The local excitation character decays monoexponentially with τ = 67 fs.

Oftentimes, the time constants obtained from fitting the adiabatic populations cannot be mapped one-to-one to the experimentally measured time constants.^72,73^ Thus, in Figure 7a, we present a simulated fluorescence spectrum, based on the energy differences and oscillator strengths between active state and ground state along all of the trajectories. Note that this spectrum was not convoluted along the time axis and thus cannot be directly compared with experimental time-resolved fluorescence spectra with a broad instrument response function. However, the simulated spectrum allows extracting a time constant for the fluorescence decay, shown in panel (b) by a monoexponential fit of the spectral data integrated between 3.4 and 5.5 eV. The resulting time constant of 85 fs is slightly longer than the lifetime of the adiabatic S_4_ state. This computed fluorescence decay constant is fully consistent with the 50–100 fs experimental time constants (τ_1_) reported previously for femtosecond fluorescence and transient absorption experiments.^19−21^ This very good agreement is a clear indication that our ADC(2) trajectories correctly represent the early deactivation cascade of *o*NBA from the initial ππ* state to the S_1_ state. Unfortunately, due to the limited length of our simulations (300 fs) and the incorrect S_1_/S_0_ crossing topology with ADC(2), the simulations do not allow pinpointing the origin of the second experimental time constant (τ_2_) of about 400 fs.^19^

We note that, interestingly, we find coherent oscillations in the emission energy as well as integrated intensity of the fluorescence spectrum (see Figure 7a,b). These oscillations have a period of approx. 25 fs. No experimental evidence of such early coherences in *o*NBA have been reported before, presumably due to the temporal resolution of previous experiments. The experimental observation of this coherent signal would provide further evidence that our picture of the early deactivation cascade of *o*NBA is accurate.

To obtain some insight into the evolution of the electronic wave function character, we computed the average CT numbers^60,74^ for the active state, shown in panel (c). Initially, the electronic state is mostly described as localized on the aromatic ring with some CT contributions, consistent with the ππ* (*L*_*b*_) state as given in Table S4. The excitation contributions localized on the ring decay with a time constant of 67 fs and exhibit slight oscillations with a period of about 25 fs. This suggests that the fluorescence signal in Figure 7b stems predominantly from the initially excited ππ* (*L*_*b*_) state. At later times, the electronic excitation localizes predominantly on the NO_2_ group, consistent with the *n*_NO_2__^–^π* character of the S_1_ minimum.

#### Nuclear Motion

**Figure 8:**
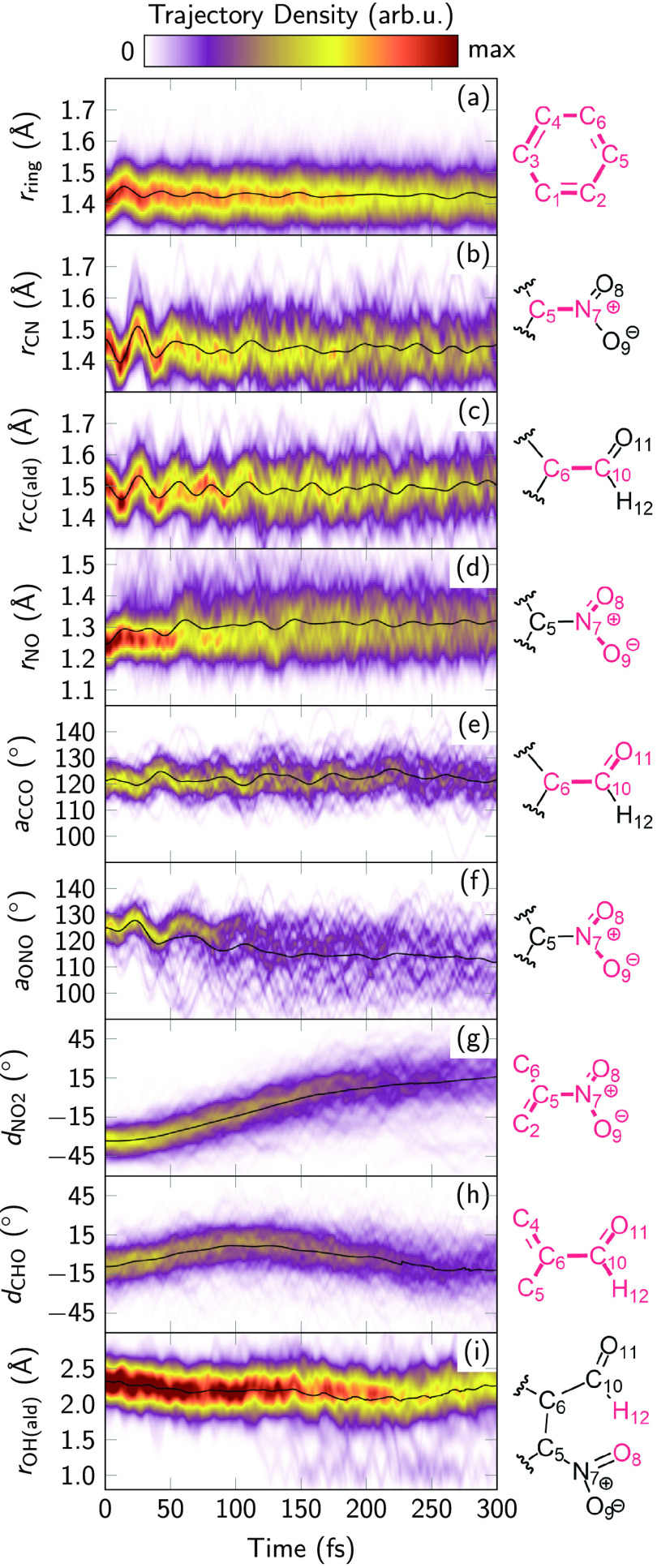
Time evolution of nuclear geometry parameters. Black lines indicate the average. (a) Average ring C–C distance. (b) C–N distance. (c) C–C_aldehyde_ distance. (d) Average N–O distance. (e) C–C–O angle. (f) O–N–O angle. (g) Average dihedral between NO_2_ group and ring. (h) Average dihedral between CHO group and ring. (i) O_NO_2__–H_aldehyde_ distance.

In Figure 8, we plot the evolution of various geometric parameters of *o*NBA. In panels (a–c), we show the average of the ring C–C distances, the C–N distances, and the distance between aromatic C and aldehyde C. All three show strongly coherent oscillations (i.e., occurring in phase in all trajectories) in the first part of the simulation. Intriguingly, these oscillations have about the same 25 fs oscillation period that we found above (Figure 7) for the fluorescence emission and excitation character (localized on the ring). Thus, it appears that excitation to the ππ* (*L*_*b*_) state induces coherent ring breathing and a strong contraction of the C–N and C–C_aldehyde_ distances that modulate emission energy and intensity. This is consistent with the bonding character of the excited π_NO_2__^*^ orbital between the C–N and C–C_aldehyde_ atoms, as shown in Figure 2. Panel (d) shows that also the N–O bonds stretch after excitation, but they do not exhibit coherent oscillations. On the contrary, the C–C–O and O–N–O bond angles (panels (e) and (f)) also exhibit similar, albeit weaker, oscillations. The O–N–O bond angle also decreases significantly while moving from the Franck–Condon region (125°) to the S_1_ minimum (115°). This is relevant because the S_1_/S_0_^NO^ crossing point is located at small O–N–O bond angles.

Figure 8g,h represents the dihedrals between the functional groups and the aromatic ring. As discussed above, some critical points including the S_1_/T_1_ minima and the S_1_/S_0_ and T_1_/S_0_ crossing points are fully planar, and consequently the initially out-of-plane functional groups (NO_2_: −33°; CHO: −10°) start to rotate after excitation. The NO_2_ group becomes planar after about 150 fs, but rotates further and reaches a dihedral of about +15° at 300 fs. The CHO group planarizes significantly faster (reaching 0° after about 50 fs), rotates slightly further, and then reverses the rotation direction. It thus seems to follow the nitro group. As shown in Figure 8i, the concerted rotations of the two functional groups shorten the distance between the aldehyde H atom and the closer nitro O atom. This prepares the molecule for HT (to form the ketene). As shown in the lower part of panel (i), a small number of trajectories indeed perform such HT, leading to O–H bond lengths of about 1 Å. The trajectories show that such short O–H distances only occur in S_1_, S_0_, or T_1_.

We present additional plots of the time evolution of geometry parameters in Figure S7 in the SI. Findings from these plots are: (i) the C=O and aldehyde C–H bonds do not oscillate notably but stretch occasionally, (ii) the six C atoms of aromatic ring stay (quite strictly) in one plane during the entire dynamics, and (iii) the nitro group becomes less rigid after excitation and can pyramidalize to some extent. Figure S8 further illustrates how rigid the aromatic ring is during the entire dynamics—the most mobile atoms are the aldehyde H and the nitro O atoms. Figure S9 demonstrates that the S_2_/S_1_^P^ crossing is the preferred MECI for the decay to S_1_, as suggested above.

In order to scrutinize the relevance of the optimized MECIs and MECPs for the actual dynamics, we performed some additional analysis. A plot of the adiabatic energies and energy gaps at all hopping events is given in Figure S10, showing that hops tend to occur at much higher energies than the MECIs/MECPs. Additionally, Figure S11 shows the distribution of the geometry parameters of the hopping points relative to the corresponding MECIs and MECPs. It can be seen that the hopping geometries are distributed rather broadly around the optimized points. This reproduces the typical finding in surface hopping trajectories that the optimized crossing points are not the places where hops actually occur due to the internal energy of the molecule, although the optimized crossings are still useful in predicting the dynamics. Note that these figures only consider the excited state–excited state crossings, as the interaction with the ground state is discussed below.

#### Decay to the Ground State

As mentioned earlier, ADC(2) is a single-reference method that does not properly represent the crossing seams between the S_0_ reference state and the excited states. In particular, this leads to very small hopping probabilities from the open-shell S_1_ to the closed-shell S_0_. Instead of hopping, most of the trajectories with small S_0_–S_1_ gaps encounter severe convergence problems, usually if the underlying Hartree–Fock becomes near-degenerate with the lowest configuration interaction singles excited state. These convergence problems typically lead to the early termination of the affected trajectory. Some trajectories instead propagate for some time in the S_1_ state at “negative” excitation energies (i.e., where the ADC(2) open-shell state is lower than the MP2 closed-shell state) before terminating. Thus, ADC(2)-based trajectories cannot provide quantitative results about the decay from S_1_ to S_0_. However, we can still obtain some qualitative understanding of the likelihood of the different decay pathways by analyzing at which geometries the trajectories stopped or where they spend more time.

Out of the 29 trajectories that terminated early, 27 stopped while their active state was the S_0_, S_1_, or T_1_ state, mostly with very small energy gaps between the S_0_ and the excited states. Only two trajectories stopped in S_2_ or T_3_ for other convergence-related reasons. Hence, generally, the trajectories first went through the relaxation cascade from the initial bright state down to S_1_ before they attempted decay to S_0_ or T_1_ and encountered convergence issues.

**Figure 9:**
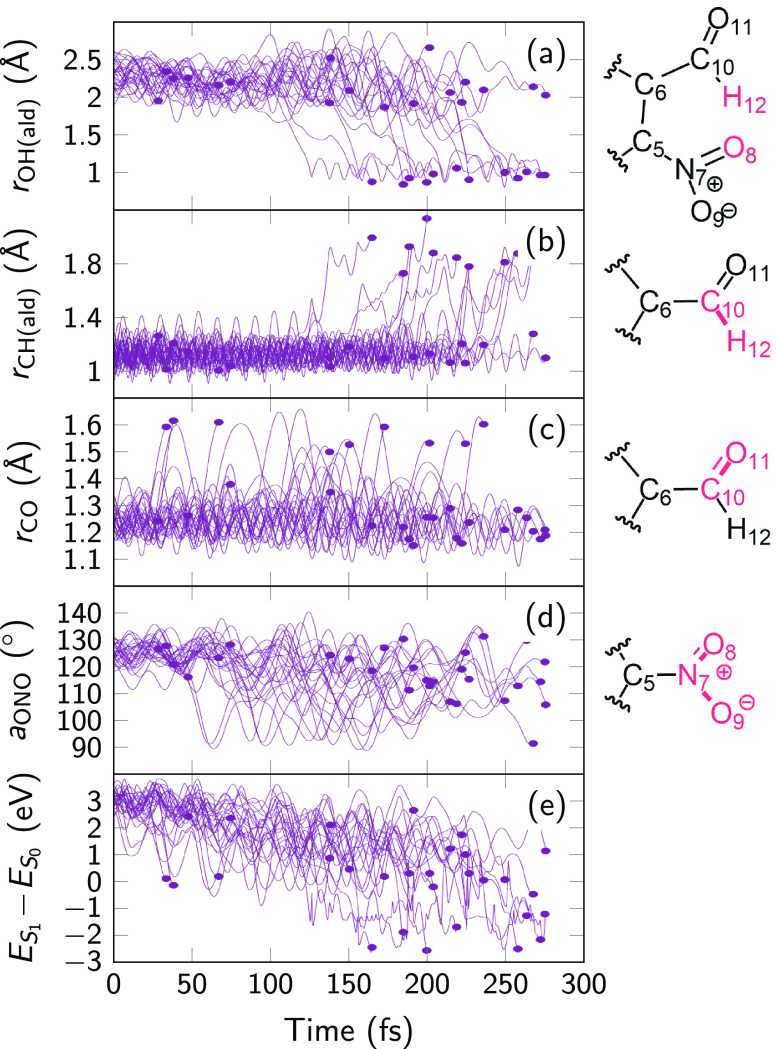
(a–d) Time evolution of the bond lengths O_8_–H_12_, C_10_–H_12_, and C_10_–O_11_ for trajectories that terminated early (i.e., did not reach 300 fs). (e) Energy gaps between S_1_ and S_0_. Dots indicate the last point of each trajectory.

In Figure 9, we show the time evolution of four important degrees of freedom for early-terminated trajectories. We plot degrees of freedom that were identified (see Tables S6 and S8) to lead to the three different S_1_/S_0_ crossing points. In panels (a) and (b), we show the distance of the aldehyde H atom to the neighboring nitro O atom and the aldehyde C atom. It can be seen that a significant number of the crashed trajectories terminated while in the process of HT (i.e., with short OH and long CH distances). Some other trajectories stopped while the carbonyl C=O bond length was significantly extended, to about 1.6 Å, as shown in panel (c). It can be seen that such long C=O bond lengths start to appear earlier than long aldehyde C–H bond lengths. Panel (d) shows that some trajectories terminated with rather small ONO bond angles. Hence, the figure shows that trajectories were terminating early either because they were attempting HT or otherwise attempting to relax to S_0_. Lastly, panel (e) presents the relevant energy gaps between S_1_ and S_0_. It can be observed that nearly all of the crashed trajectories exhibit energy gaps that are very small or negative, as mentioned above. The early-terminated trajectories showed that there are only three crossing regions with the S_0_—indicated with the superscripts CO, NO, and HT—and our PES exploration did not miss any relevant crossing region.

**Figure 10:**
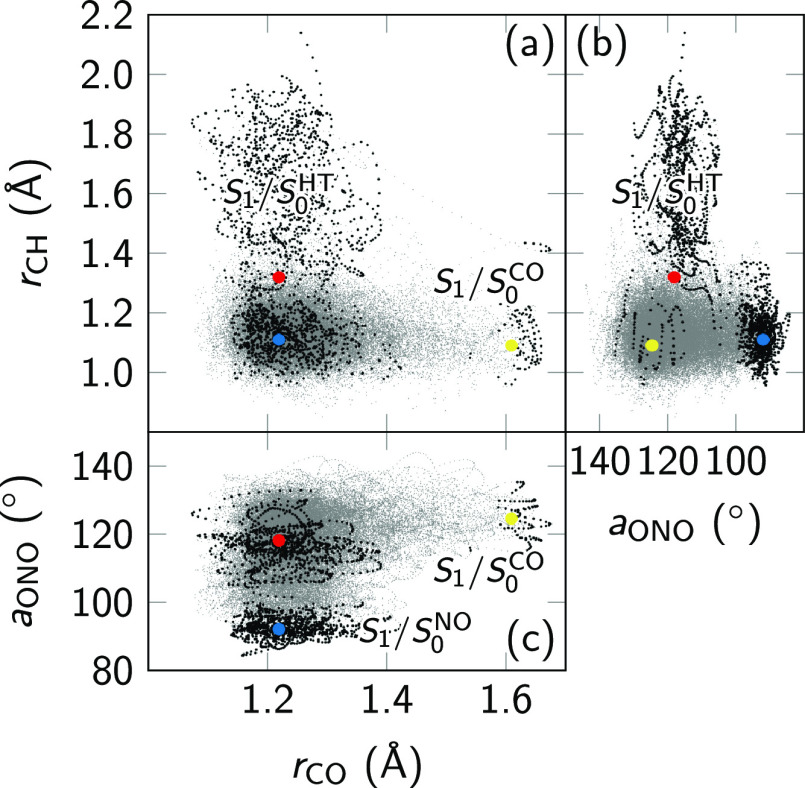
(a–c) Scatter plots of the C_10_–O_11_ and C_10_–H_12_ bond lengths and the O_8_–N_7_–O_9_ bond angle. Gray dots show the distribution of the entire swarm of 96 trajectories over all time steps. Black dots are all time steps where the S_1_ energy is below the S_0_ for any of those trajectories. Colored dots indicate the position of the S_1_/S_0_^NO^ (blue) and S_1_/S_0_^CO^ (yellow) and S_1_/S_0_^HT^ (red) crossing points optimized with ADC(2).

Investigating the early-terminated trajectories is a useful strategy to identify potential decay pathways, but it is less useful to estimate the relative importance of the decay pathways. As ADC(2) does not describe the decay to the S_0_ correctly, we provide an estimation of the relative importance of the decay channels based on how often regions of zero/negative S_1_–S_0_ gap are visited. Figure 10 shows a scatter plot of the C_10_–O_11_ and C_10_–H_12_ bond lengths as well as the O_8_–N_7_–O_9_ bond angle. Faint gray dots show the full distribution of the swarm of trajectories over all time steps. Stronger black dots mark those geometries where the S_1_–S_0_ gap is zero or negative, and decay to S_0_ might be very likely.

There are three clearly distinguishable clusters of such geometries. The first cluster can be identified by an aldehyde C–H bond that is longer than approx. 1.3 Å—hence, this cluster is related to the S_1_/S_0_^HT^ decay channel. 47% of all black dots fulfill this bond length criterion. Notably, the termination points have much longer C–H bond lengths than the S_1_/S_0_^HT^ crossing point, indicating that ADC(2) can actually describe the first part of the HT process. A second cluster is found for ONO angles below 100°, located close to the S_1_/S_0_^NO^ crossing point and accounting for another 47% of all points with negative S_1_–S_0_ gaps. The third—much smaller cluster—is distinguished by C=O bond lengths that are longer than 1.6 Å. This cluster can be assigned to the S_1_/S_0_^CO^ crossing point and includes 4% of the black dots. The remaining 2% of black dots are not captured by these simple thresholds because they fall in between the clusters. Note that the trajectories undergoing ISC are included in these percentages.

The relative occurrence of the three negative-energy regions in the trajectories matches well with the information that we have obtained from the optimizations. At ADC(2) level of theory, both the S_1_/S_0_^HT^ and S_1_/S_0_^NO^ crossing points are easily accessible from the S_1_ minimum, with barriers of about 0.1 and 0.2 eV, respectively. On the contrary, the S_1_/S_0_^CO^ crossing point is located much higher, with a barrier of about 0.5 eV. These barriers roughly explain why the latter crossing point is much less populated than the S_1_/S_0_^HT^ and S_1_/S_0_^NO^ crossing points. Moreover, our MS-CASPT2 calculations showed that ADC(2) severely underestimates the energy of the S_1_/S_0_^CO^ crossing point, which should be about 2 eV above the S_1_ minimum. Consequently, this crossing point should be excluded from our analysis. The remaining data in our simulations then shows that there should be two decay pathways in *o*NBA with roughly equal likelihood. These are (i) the decay back to the ground state via the S_1_/S_0_^NO^ crossing point that involves a scissoring motion of the nitro group and (ii) the transfer of the aldehyde H atom to the nitro group to form the ketene intermediate. The ratio between these paths that we obtain is 47:47, which explains the experimental value of approximately 50% for the quantum yield of nitrosobenzoic acid.

### Photophysics of *o*NBA

**Figure 11:**
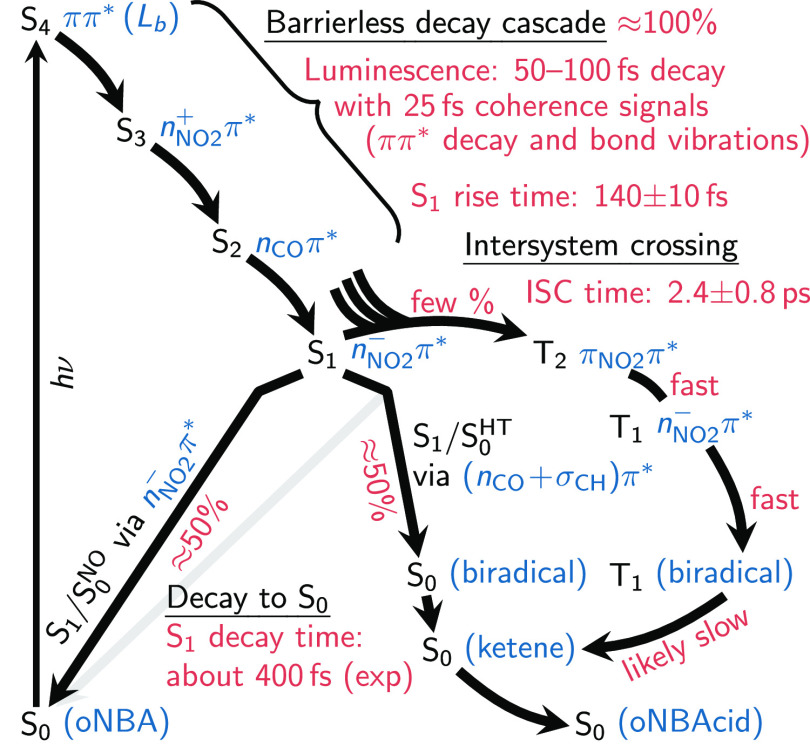
Overview scheme over the likely photophysics of *o*NBA after excitation to the ππ* (*L*_*b*_) state.

The findings regarding the photophysics of *o*NBA from the present work are summarized in Figure 11, divided into three main processes.

The nonadiabatic dynamics of oNBA is initiated by the vertical excitation to the ππ* (*L*_*b*_) state—which is the lowest-energy ππ* state (although it is not particularly bright)—or a higher state. This excitation first launches an ultrafast, barrierless singlet decay cascade of the molecule via the *n*_NO_2__^+^π* and *n*_CO_π* states to the minimum of the *n*_NO_2__^–^π* state on the S_1_ adiabatic potential energy surface. Our dynamics simulations show that this process occurs with near-unity yield, although some ISC might occur already on the way to the S_1_ minimum. The decay can be observed with time-resolved luminescence spectroscopy, where we find an 85 fs decay of the ππ* emission, compared to a value of 100 fs from experiment (τ_1_).^19,21^ Furthermore, we predict a short-lived coherent oscillation in the fluorescence energy with a period of about 25 fs that has not been reported before to the best of our knowledge. The oscillations arise from coherent bond vibrations induced by populating the ππ* state and vanish within the lifetime of that state. The decay cascade leads to a rise time of the S_1_ population of about 140 fs.

The second significant process is ISC. Our dynamics simulations predict an ISC yield of at about 9% after 300 fs and an ISC time constant of 2.4 ± 0.8 ps, providing computational confirmation of the experimental estimate of 2.7 ps.^27^ ISC was found to take place to some extent during the S_4_ → S_1_ cascade as well as from the S_1_, although our simulations do not allow pinpointing the predominant ISC channel due to the small number of ISC events. Large SOC matrix elements are only found among the singlet and triplet excitations localized on the nitro group, so it is possible that the population of the low-lying aldehyde *n*_CO_π* state slightly slows down ISC compared to nitrobenzene. We expect that the triplet state involved in ISC is the ^3^π_NO_2__π* state (the ππ* state localized on the nitro group), from which the lower ^3^*n*_NO_2__^–^π* is immediately reached. However, only a small barrier needs to be overcome to leave the ^3^*n*_NO_2__^–^π* minimum and either access T_1_/S_0_ crossings or undergo HT in the T_1_. This HT in the triplet state leads to a biradical intermediate that is relatively long-lived because of very small SOCs (0.5 cm^–1^), but eventually undergoes ISC back to the S_0_, forming the closed-shell ketene. This slow process was already previously suggested^20^ to explain the delayed rise time of the ketene of about 200 ps (τ_4_).

The third important decay process is the direct IC from the S_1_ minimum back to the S_0_. We identified three potential MECIs, but the S_1_/S_0_^CO^ intersection is not energetically feasible according to MS-CASPT2. The two remaining MECIs are the S_1_/S_0_^NO^ and S_1_/S_0_^HT^ intersections. The S_1_/S_0_^NO^ intersection is reached by a scissoring motion of the nitro group, exhibits a barrier of about 0.2–0.3 eV, and leads back to the oNBA ground state with near-unity efficiency due to the sloped shape of the conical intersection. The S_1_/S_0_^HT^ intersection is due to HT from the aldehyde group to the nitro group. The simulations show that such HT can only occur in S_1_, S_0_, or T_1_, but not in higher states. A barrier of 0.1–0.2 eV has to be overcome for HT, which is slightly smaller than for S_1_/S_0_^NO^, but according to the dynamics simulations, the S_1_/S_0_^NO^ and S_1_/S_0_^HT^ regions are visited with approximately equal probabilities. From the S_1_/S_0_^HT^ crossing, we expect that a significant fraction of the trajectories continue with HT,^25^ form the biradical structure and eventually the ketene. However, the peaked shape of the S_1_/S_0_^HT^ MECI might allow some fraction of the molecules to return back to the oNBA ground state.

## Conclusions

In this work, we present an extensive theoretical study on the excited state relaxation mechanisms of *ortho*-nitrobenzaldehyde (*o*NBA), with focus on the initial internal conversion, the possibility of intersystem crossing, and the formation of the ketene intermediate. We employed two electronic structure methods: ADC(2), a single-reference method that enables us to efficiently explore the excited state potential energy surfaces, and MS-CASPT2, a multireference method that provides expensive yet reliable results where ADC(2) is unreliable. The two methods agree very well, except for the higher-lying excited states and for one deactivation side channel.

Our results are summarized in full detail in Figure 11 and the Photophysics of oNBA Section. We fully characterized the decay cascade from the initially excited ππ* (*L*_*b*_) state (S_4_) to the *n*_NO_2__^–^π* minimum (S_1_), providing information on all relevant conical intersections (CIs), involved electronic states, deactivation time scales, and expected spectroscopic signals. From the S_1_ minimum, we characterized both intersystem crossing and internal conversion. Intersystem crossing can occur with low probability either during the initial decay cascade or from the S_1_ minimum, but is significantly slower than internal conversion processes. Once in the triplet state, the molecule quickly reaches the T_1_ minimum of oNBA and from there forms a long-lived biradical structure via HT. Eventually, the triplet crosses back to a singlet state and then forms a ketene. The investigation of the internal conversion pathways from the S_1_ minimum showed that two CIs are responsible for the decay. One CI that involves a scissoring motion of the nitro group leads to the recovery of the *o*NBA ground state. The other CI is connected with HT from the aldehyde to the nitro group. It likely leads to the formation of the ketene intermediate, but we cannot exclude that some fraction of HTs are reversed and lead back to the *o*NBA ground state. The two CIs will be activated with roughly equal probability, explaining the 40–50% quantum yield of ketene formation.^75^

## Supporting Information Available

Additional details on the basis set comparison and optimization settings; results on vertical excitation calculations; optimized geometries; potential energy surfaces and dynamics; and cartesian coordinates of all optimized geometries (PDF)
