# Supplementary material for: Role of Ultrafast Internal Conversion and Intersystem Crossing in the Nonadiabatic Relaxation Dynamics of ortho-Nitrobenzaldehyde
Source: J Phys Chem A. 2023 Jul 5;127(28):5872–86. doi: 10.1021/acs.jpca.3c02899 (PMC10364085; doi:10.1021/acs.jpca.3c02899)
Supplement: Supplementary file 1 — jp3c02899_si_001.pdf [file jp3c02899_si_001.pdf]

Supporting Information: The Role of Ultrafast Internal Conversion and Intersystem Crossing in the Nonadiabatic Relaxation Dynamics of Ortho-nitrobenzaldehyde

Dóra Vörös[†,‡] and Sebastian Mai[*,†]

†*Institute of Theoretical Chemistry, Faculty of Chemistry, University of Vienna, Währinger Str. 17, 1090 Vienna, Austria*

‡*Vienna Doctoral School in Physics, University of Vienna, Boltzmanngasse 5, 1090 Vienna, Austria.*

E-mail: sebastian.mai@univie.ac.at

Contents

S1 Computational Details **S-2**

S1.1 Basis Set Comparison S-2

S1.2 Optimization Settings S-3

S1.3 Wigner distribution S-4

S2 Vertical Excitation Calculations **S-5**

S3 Supplementary optimization results **S-7**

S4 Excited-State Potential Energy Surfaces **S-14**

S5 Dynamics **S-15**

S6 Coordinates **S-19**

S1 Computational Details

S1.1 Basis Set Comparison

Tables S1 and S2 compare vertical excitation calculations using the cc-pVTZ basis set—as used in our previous benchmark study—and the cc-pVDZ basis set, which was employed in all optimizations and the dynamics simulations. For ADC(2) (Table S1), reducing the basis set from triple-ζ to double-ζ leads to a blue-shift of all excitation energies, with smaller shifts for the low-lying states (0.06–0.09 eV for S_1 to S_3) and slightly larger shifts for the higher-lying states (0.12–0.19 eV for S_4 to S_6). The triplet states are also slightly blue-shifted. Oscillator strengths and state characters are virtually unaffected by the change of basis set.

Similar findings apply to MS-CASPT2 (Table S2), with the exception that the S_5 state is shifted by 0.4 eV. Nonetheless, based on these results, we expect that the smaller cc-pVDZ basis set is adequate for the optimizations performed in this work.

Table S1: Effect of the basis set on vertical excitation energies at ADC(2) level of theory.

State	cc-pVDZ			cc-pVTZ		
	ΔE	f_{osc}	Character	ΔE	f_{osc}	Character
S_1	3.54	0.001	$n_{\mathrm{CO}}\pi^*_{NO2}$ (local)	3.48	0.001	$n_{\mathrm{CO}}\pi^*_{NO2}$ (local)
S_2	3.71	0.008	$n^-_{\mathrm{NO2}}\pi^*$ (local)	3.63	0.008	$n^-_{\mathrm{NO2}}\pi^*$ (local)
S_3	4.26	0.001	$n^+_{\mathrm{NO2}}\pi^*$ (local)	4.17	0.001	$n^+_{\mathrm{NO2}}\pi^*$ (local)
S_4	4.78	0.021	$\pi\pi^*$ (L_b)	4.66	0.024	$\pi\pi^*$ (L_b)
S_5	5.32	0.004	$n_{\mathrm{CO}}\pi^*$ (CT)	5.14	0.006	$n_{\mathrm{CO}}\pi^*$ (CT)
S_6	5.61	0.151	$\pi\pi^*$ (L_a)	5.42	0.149	$\pi\pi^*$ (L_a)
T_1	3.16	—	$n_{\mathrm{CO}}\pi^*$ (local)	3.14	—	$n_{\mathrm{CO}}\pi^*$ (local)
T_2	3.36	—	$n^-_{\mathrm{NO2}}\pi^*/\pi\pi^*$ (L_a)	3.31	—	$n^-_{\mathrm{NO2}}\pi^*/\pi\pi^*$ (L_a)
T_3	3.64	—	$\pi\pi^*$ (NO_2)	3.67	—	$\pi\pi^*$ (NO_2)
T_4	3.93	—	$n^+_{\mathrm{NO2}}\pi^*/\pi\pi^*$ (L_a)	3.87	—	$n^+_{\mathrm{NO2}}\pi^*/\pi\pi^*$ (L_a)

Table S2: Effect of the basis set on vertical excitation energies at MS-CASPT2(18,14) level of theory.

State	cc-pVDZ			cc-pVTZ		
	ΔE	f_{osc}	Character	ΔE	f_{osc}	Character
S_1	3.73	0.001	$n_{\mathrm{CO}}\pi^*$ (local)	3.58	0.001	$n_{\mathrm{CO}}\pi^*$ (local)
S_2	3.82	0.003	$n^-_{\mathrm{NO2}}\pi^*$ (local)	3.77	0.004	$n^-_{\mathrm{NO2}}\pi^*$ (local)
S_3	4.28	0.001	$n^+_{\mathrm{NO2}}\pi^*$ (local)	4.20	0.001	$n^+_{\mathrm{NO2}}\pi^*$ (local)
S_4	4.66	0.003	$\pi\pi^*$ (L_b)	4.54	0.003	$\pi\pi^*$ (L_b)
S_5	5.99	0.052	$\pi\pi^*$ (L_a), dex	5.58	0.058	$\pi\pi^*$ (L_a), dex
S_6	6.23	0.003	dex, $n_{\mathrm{CO}}\pi^*$ (CT)	6.10	0.001	dex, $n_{\mathrm{CO}}\pi^*$ (CT)
T_1	3.40	—	$n_{\mathrm{CO}}\pi^*$ (local)	3.37	—	$n_{\mathrm{CO}}\pi^*$ (local)
T_2	3.49	—	$\pi\pi^*$ (NO_2)	3.49	—	$\pi\pi^*$ (NO_2)
T_3	3.60	—	$n^-_{\mathrm{NO2}}\pi^*$ and $\pi\pi^*$ (L_a)	3.53	—	$n^-_{\mathrm{NO2}}\pi^*$ and $\pi\pi^*$ (L_a)
T_4	3.75	—	$n^+_{\mathrm{NO2}}\pi^*$ and $\pi\pi^*$ (L_a)	3.70	—	$n^+_{\mathrm{NO2}}\pi^*$ and $\pi\pi^*$ (L_a)

S1.2 Optimization Settings

Table S3 summarizes the different critical points on the potential energy surfaces that were optimized and the methods used for these optimizations. As mentioned in the main text, S_5/S_4 was not optimized with ADC(2) because the S_4 is the bright state at this level of theory. Moreover, S_0^{bir}min and T_1^{bir}min were not successfully optimized with ADC(2) because the wave function acquires strong multi-reference character close to the ketene transformation point. The S_4/S_3, S_3/S_2, and S_2/S_1^P MECIs were not optimized with MS-CASPT2 because ADC(2)-optimized geometries were found to already give very small energy gaps at MS-CASPT2 level. In addition, S_1/T_2 and T_2/T_1 MECPs were taken from a preliminary MS-CASPT2 scan because the S_1/T_2 and T_2/T_1 geometries optimized with a smaller active space were not yielding reasonable results in the calculations with bigger active space.

Table S3: Overview over considered critical points and methods used for optimization.

Label	Geometry	ADC(2)[a]	MS-CASPT2[b]
1	S_0min	MP2	MP2
2	S_5/S_4	—	MS(12,0)-CASPT2(12,9)
3	S_4/S_3	ADC(2)	Taken from ADC(2)
4	S_3/S_2	ADC(2)	Taken from ADC(2)
5	S_2/S_1^P	ADC(2)	Taken from ADC(2)
6	S_2/S_1	ADC(2)	—
7	S_1min	ADC(2)	MS(4,0)-CASPT2(12,9)
8	S_1/S_0^{CO}	ADC(2)	MS(4,0)-CASPT2(12,10)
9	S_1/S_0^{NO}	ADC(2)	MS(4,0)-CASPT2(12,10)
10	S_1/S_0^{HT}	ADC(2)	MS(6,0)-CASPT2(12,10)
11	S_0^{bir}min	—	MS(3,0)-CASPT2(12,9)
12	S_1/T_2^P	ADC(2)	—
13	S_1/T_2	ADC(2)	Taken from scan between ADC(2) geometries 12 and 14
14	T_2/T_1	ADC(2)	Taken from scan between ADC(2) geometries 12 and 14
15	T_1min	ADC(2)	MS(0,3)-CASPT2(12,9)
16	T_1/S_0^{CO}	ADC(2)	MS(4,3)-CASPT2(12,10)
17	T_1/S_0^{NO}	ADC(2)	MS(4,3)-CASPT2(12,10)
18	T_1/S_0^{HT}	ADC(2)	MS(4,3)-CASPT2(12,10)
19	T_1^{bir}min	—	MS(0,3)-CASPT2(12,10)

[a] All optimizations with cc-pVDZ and default auxiliary basis set.

[b] All optimizations with cc-pVDZ, default IPEA shift [0.25], imaginary shift of 0.1 a.u., and Cholesky decomposition.

S1.3 Wigner distribution

We generated 1000 initial conditions from the Wigner distribution of the ground state minimum and used those 1000 geometries for computing the absorption spectrum. For the nonadiabatic trajectories, we selected 96 of those initial conditions based on the oscillator strengths at these geometries. Figure S1 shows that the 96 selected initial conditions provide sufficiently smooth distributions, showing that the subset of initial conditions represents the Wigner distribution adequately. Note that the selection of the initial conditions depends on the oscillator strengths, so deviations form the expected Gaussian distribution can arise from the geometry dependence of the oscillator strengths.

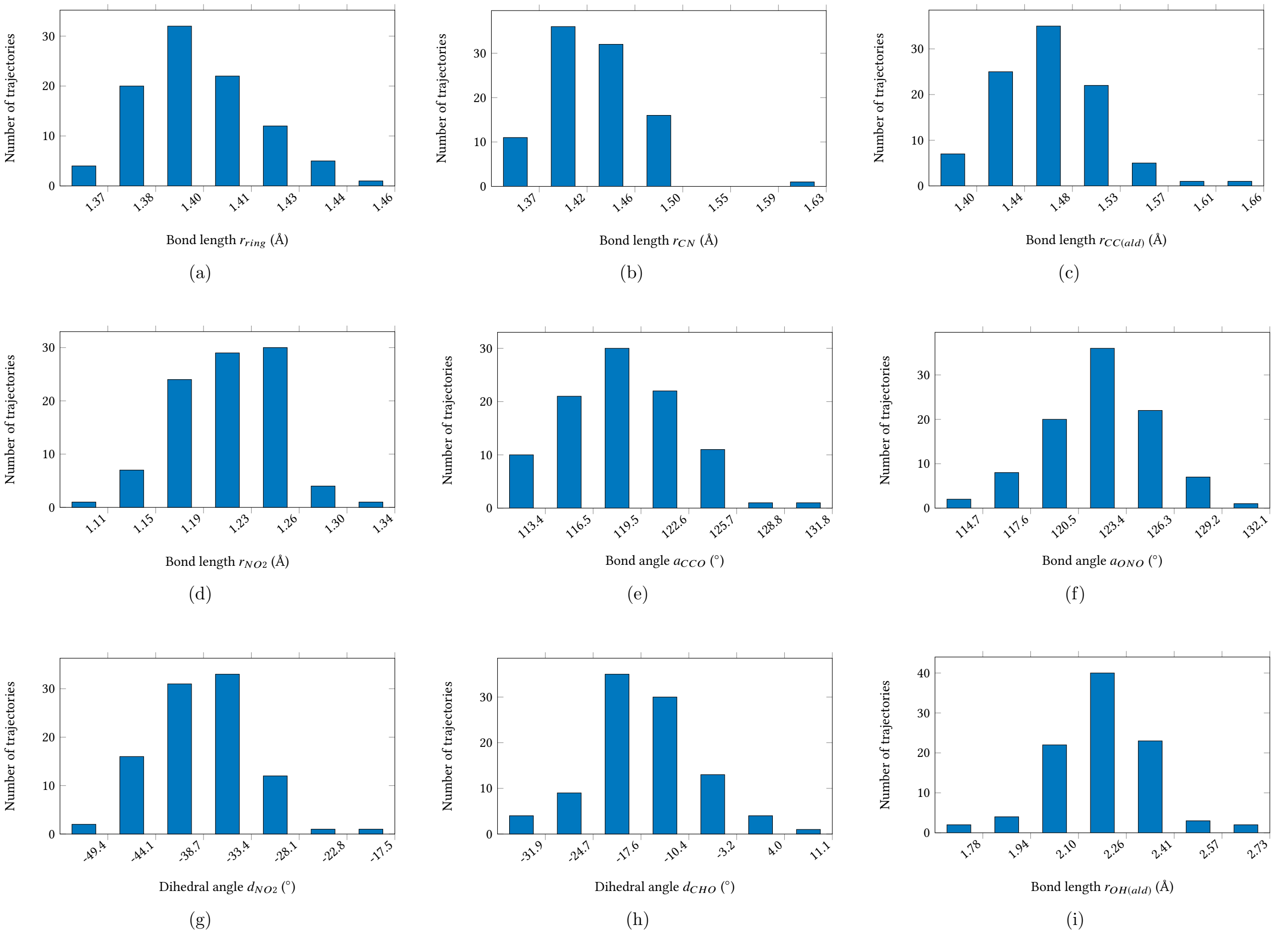

Figure S1: Histograms showing the distribution of several geometric parameters at $t = 0$ for the 96 initial conditions from which the trajectories were generated. The geometric parameters are defined and described in the main text related to Figure 8.

S2 Vertical Excitation Calculations

Table S4: Vertical excitation energies of oNBA, computed using ADC(2)/cc-pVTZ and MS(12)-CASPT2(18,14)/cc-pVTZ.

State	E	f_{osc}	Ω	Ω_{ring}	Ω_{NO_2}	Ω_{CHO}	CT	Char.
				— ADC(2)/cc-pVTZ —				
S_1	3.48	0.001	0.91	0.03	0.07	0.43	0.37	$n_{CO}\pi^*$ (local)
S_2	3.63	0.008	0.90	0.02	0.61	0.06	0.22	$n^-_{NO2}\pi^*$ (local)
S_3	4.17	0.001	0.90	0.03	0.62	0.01	0.24	$n^+_{NO2}\pi^*$ (local)
S_4	4.66	0.024	0.89	0.58	0.03	0.00	0.28	$\pi\pi^*$ (L_b)
S_5	5.14	0.006	0.85	0.07	0.04	0.05	0.69	$n_{CO}\pi^*$ (CT)
S_6	5.42	0.149	0.90	0.36	0.06	0.03	0.45	$\pi\pi^*$ (L_a)
S_7	5.82	0.109	0.87	0.18	0.13	0.00	0.56	$\pi\pi^*$ (B_a/B_b, NO_2), CT
S_8	6.05	0.287	0.88	0.13	0.30	0.01	0.44	$\pi\pi^*$ (B_a/B_b, NO_2)
S_9	6.08	0.206	0.88	0.27	0.15	0.02	0.46	$\pi\pi^*$ (B_a/B_b, NO_2)
S_{10}	6.31	0.169	0.86	0.19	0.06	0.02	0.59	$\pi\pi^*$ (B_a/B_b), CT
S_{11}	6.36	0.005	0.85	0.15	0.04	0.02	0.63	$\pi\pi^*$ (B_a/B_b), CT
T_1	3.14	—	0.92	0.03	0.04	0.49	0.35	$n_{CO}\pi^*$ (local)
T_2	3.31	—	0.91	0.08	0.58	0.03	0.22	$n^-_{NO2}\pi^*$ and $\pi\pi^*$ (L_a)
T_3	3.67	—	0.94	0.00	0.77	0.01	0.16	$\pi\pi^*$ (NO_2)
T_4	3.87	—	0.93	0.44	0.21	0.04	0.24	$n^+_{NO2}\pi^*$ and $\pi\pi^*$ (L_a)
				— MS(12)-CASPT2(18,14)/cc-pVTZ —				
S_1	3.58	0.001	0.77	0.01	0.12	0.49	0.16	$n_{CO}\pi^*$ (local)
S_2	3.77	0.004	0.77	0.01	0.55	0.10	0.10	$n^-_{NO2}\pi^*$ (local)
S_3	4.20	0.001	0.74	0.09	0.53	0.00	0.11	$n^+_{NO2}\pi^*$ (local)
S_4	4.54	0.003	0.72	0.52	0.10	0.00	0.10	$\pi\pi^*$ (L_b)
S_5	5.58	0.058	0.53	0.28	0.02	0.01	0.22	$\pi\pi^*$ (L_a), dex
S_6	6.10	0.001	0.24	0.01	0.01	0.02	0.20	dex, $n_{CO}\pi^*$ (CT)
S_7	6.38	0.015	0.20	0.00	0.04	0.00	0.16	dex, $\pi\pi^*$ (NO_2)
S_8	6.67	0.001	0.21	0.01	0.00	0.09	0.11	dex, CT
S_9	6.75	0.009	0.19	0.03	0.02	0.01	0.13	dex, CT
S_{10}	6.92	0.000	0.00	0.00	0.00	0.00	0.00	dex
S_{11}	7.22	0.003	0.14	0.00	0.01	0.00	0.13	dex, CT
T_1	3.37	—	0.81	0.02	0.01	0.64	0.15	$n_{CO}\pi^*$ (local)
T_2	3.49	—	0.93	0.03	0.83	0.00	0.07	$\pi\pi^*$ (NO_2)
T_3	3.53	—	0.86	0.17	0.59	0.01	0.09	$n^-_{NO2}\pi^*$ and $\pi\pi^*$ (L_a)
T_4	3.70	—	0.94	0.68	0.10	0.06	0.09	$n^+_{NO2}\pi^*$ and $\pi\pi^*$ (L_a)

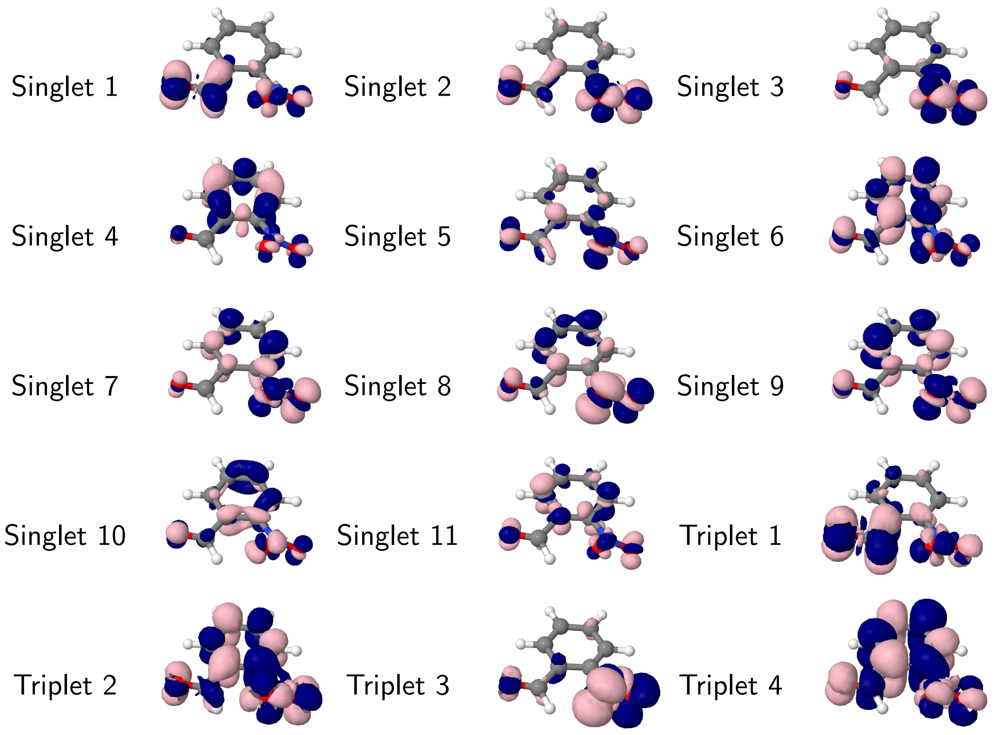

Figure S2: Transition densities for the excited states computed with ADC(2)/cc-pVDZ.

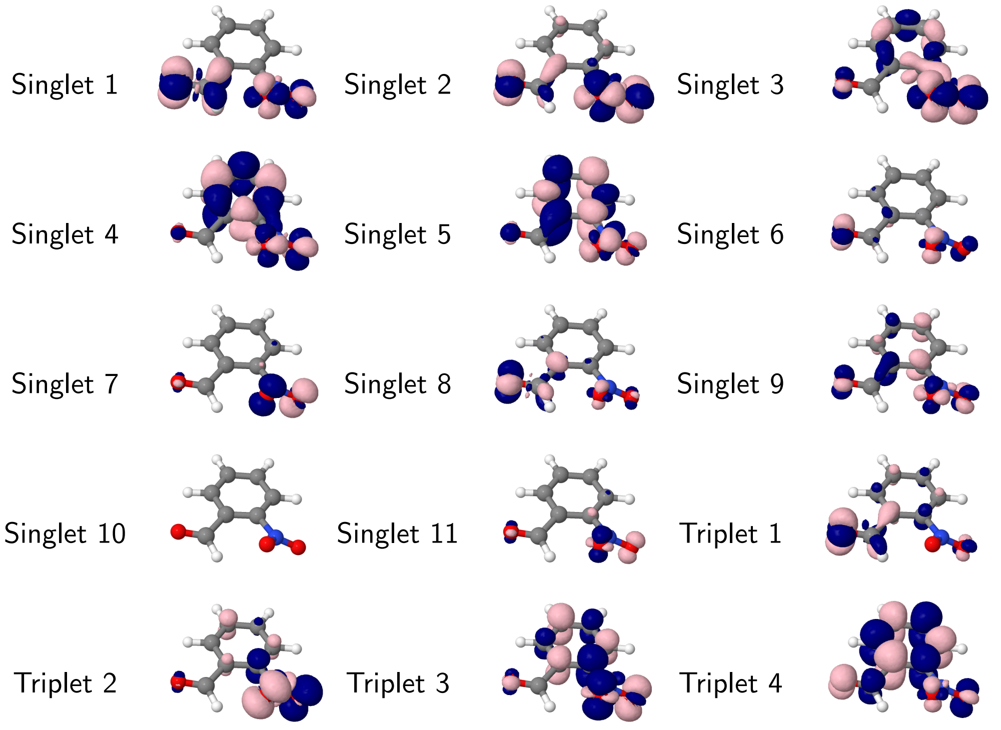

Figure S3: Transition densities for the excited states computed with MS-CASPT2(18,14)/cc-pVTZ.

S3 Supplementary optimization results

Table S5 and Table S7 contain the relative energies and characters of the optimized geometry calculated at ADC(2) and MS-CASPT2 levels of theory, respectively.

Table S5: ADC(2)/cc-pVDZ excited-state energies[a] at the critical points optimized with ADC(2)/cc-pVDZ (cf. Table S3).

Label	Geom.[b]	Character[c]	S_0	S_1	S_2	S_3	S_4	S_5	T_1	T_2	T_3	SOC[d]
1	S_0min	GS	**0.00**	3.54	3.71	4.26	**4.78**	5.32	3.16	3.36	3.64	
2	S_5/S_4	(not computed with ADC(2))	—	—	—	—	—	—	—	—	—	
3	S_4/S_3	$\pi\pi^*$ (L_b) and $n^+_{\mathrm{NO2}}\pi^*$	0.51	3.53	3.87	**4.46**	**4.51**	5.34	3.21	3.58	3.78	
4	S_3/S_2	$n^+_{\mathrm{NO2}}\pi^*$ and $n_{\mathrm{CO}}\pi^*$	0.46	3.32	**3.72**	**3.79**	5.02	5.09	3.04	3.25	3.38	
5	S_2/S_1^P	$n^-_{\mathrm{NO2}}\pi^*$ and $n_{\mathrm{CO}}\pi^*$	0.43	**3.18**	**3.28**	4.29	4.94	5.08	2.86	2.97	3.34	
6	S_2/S_1	$n^-_{\mathrm{NO2}}\pi^*$ and $n^+_{\mathrm{NO2}}\pi^*$	1.19	**3.63**	**3.70**	4.80	5.50	5.70	3.34	3.37	3.53	
7	S_1min	$n^-_{\mathrm{NO2}}\pi^*$	1.25	**2.56**	4.24	4.75	4.97	5.65	2.36	3.19	3.94	
8	S_1/S_0^{CO}	$n_{\mathrm{CO}}\pi^*$ and GS	**3.07**	**3.15**	5.33	5.45	6.42	6.81	3.03	4.14	5.46	
9	S_1/S_0^{NO}	$n^-_{\mathrm{NO2}}\pi^*$ and GS	**2.70**	**2.77**	5.42	5.45	5.81	6.10	2.58	3.91	5.23	
10	S_1/S_0^{HT}	$(n_{\mathrm{CO}}+\sigma_{\mathrm{CH}})\pi^*$ and GS	**2.42**	**2.45**	4.39	5.73	6.00	6.24	2.32	4.13	5.37	
11	S_0^{bir}min	(not computed with ADC(2))	—	—	—	—	—	—	—	—	—	
12	S_1/T_2^P	$^1n^-_{\mathrm{NO2}}\pi^*$ and $^3n_{\mathrm{CO}}\pi^*$	0.44	**2.95**	3.29	4.21	4.88	5.02	2.68	**2.95**	3.23	7
13	S_1/T_2	$^1n^-_{\mathrm{NO2}}\pi^*$ and $^3\pi_{\mathrm{NO2}}\pi^*$	0.86	**3.03**	3.79	4.40	5.23	5.39	2.73	**3.03**	3.52	50
14	T_2/T_1	$n^-_{\mathrm{NO2}}\pi^*$ and $n_{\mathrm{CO}}\pi^*$	0.37	3.17	3.29	4.26	4.91	5.08	**2.86**	**2.95**	3.30	
15	T_1min	$n^-_{\mathrm{NO2}}\pi^*$	1.35	2.61	4.44	4.78	5.16	5.63	**2.35**	3.18	4.15	
16	T_1/S_0^{CO}	$^3n_{\mathrm{CO}}\pi^*$ and GS	**3.00**	3.14	5.40	5.43	6.35	6.81	**3.00**	4.14	5.40	—
17	T_1/S_0^{NO}	$^3n^-_{\mathrm{NO2}}\pi^*$ and GS	**2.43**	2.76	5.26	5.69	5.76	6.24	**2.43**	3.50	5.01	—
18	T_1/S_0^{HT}	$^3(n_{\mathrm{CO}}+\sigma_{\mathrm{CH}})\pi^*_{NO2}$ and GS	**2.33**	2.46	4.37	5.67	5.97	6.21	**2.32**	4.11	5.25	—
19	T_1^{bir}min	(not computed with ADC(2))	—	—	—	—	—	—	—	—	—	

[a] All energies are in eV relative to the S_0 MP2 energy at the S_0min geometry. [b] See Table S3 for optimization settings. [c] According to transition densities. [d] In cm^{-1}. Turbomole cannot compute SOCs between S_0 and triplets.

Table S6: Geometry parameters[a] (bond lengths r, bond angles a, and dihedrals d) and SOCs for the excited-state minima and crossing points optimized at ADC(2)/cc-pVDZ level of theory.

Label	Geom.	$r_{8,12}$ (Å)	$r_{9,13}$ (Å)	$r_{10,12}$ (Å)	$r_{10,11}$ (Å)	$r_{7,5}$ (Å)	$r_{6,10}$ (Å)	$a_{12,10,6}$ (°)	$a_{11,10,6}$ (°)	$a_{8,7,9}$ (°)	$d_{6,5,7,8}$ (°)	$d_{12,10,6,5}$ (°)	$p_{5,7,8,9}$ (°)
1	S_0min	2.29	2.42	1.11	1.22	1.48	1.51	117.0	121.5	125.8	**-35**	**-8**	1
2	S_5/S_4	—	—	—	—	—	—	—	—	—	—	—	—
3	S_4/S_3	2.24	2.35	1.11	1.24	1.43	1.48	116.9	120.4	124.7	-8	-4	1
4	S_3/S_2	2.31	2.44	1.11	1.25	1.45	1.48	118.4	121.5	127.9	-44	-6	14
5	S_2/S_1^P	2.35	2.42	1.10	1.30	1.43	1.45	121.2	122.1	119.1	-35	-2	1
6	S_2/S_1	2.40	2.46	1.11	1.22	1.50	1.50	116.2	122.0	131.4	-63	0	39
7	S_1min	2.10	2.44	1.11	1.23	**1.38**	1.49	116.3	122.5	**104.7**	**0**	**0**	**0**
8	S_1/S_0^{CO}	2.26	**2.21**	1.09	**1.61**	1.46	**1.37**	**136.7**	**116.4**	124.6	0	0	0
9	S_1/S_0^{NO}	2.19	2.46	1.11	1.23	**1.35**	1.49	116.3	122.7	**94.1**	3	0	-2
10	S_1/S_0^{HT}	**1.24**	2.73	**1.32**	1.22	1.41	1.46	**100.9**	**130.9**	118.1	0	0	0
11	S_0^{bir}min	—	—	—	—	—	—	—	—	—	—	—	—
12	S_1/T_2^P	2.15	2.42	1.10	1.28	1.42	1.45	120.1	122.6	116.9	-19	0	0
13	S_1/T_2	2.48	2.47	1.11	1.22	1.46	1.50	116.4	122.6	119.9	-52	-12	**32**
14	T_2/T_1	2.34	2.44	1.10	1.29	1.43	1.45	121.1	121.7	118.7	-37	-2	-1
15	T_1min	2.18	2.44	1.11	1.22	1.41	1.50	116.4	122.3	**104.2**	**-29**	**-3**	**19**
16	T_1/S_0^{CO}	2.26	**2.23**	1.09	**1.61**	1.46	**1.37**	**138.3**	**116.0**	124.5	0	0	0
17	T_1/S_0^{NO}	2.35	2.49	1.11	1.22	1.44	1.50	116.1	122.5	**97.8**	-56	-2	37
18	T_1/S_0^{HT}	**1.26**	2.76	**1.29**	1.22	1.40	1.45	**101.5**	**131.8**	118.0	0	0	0
19	T_1^{bir}min	—	—	—	—	—	—	—	—	—	—	—	—

[a] The numbering of the atoms is introduced in Fig. 1 in the main text.

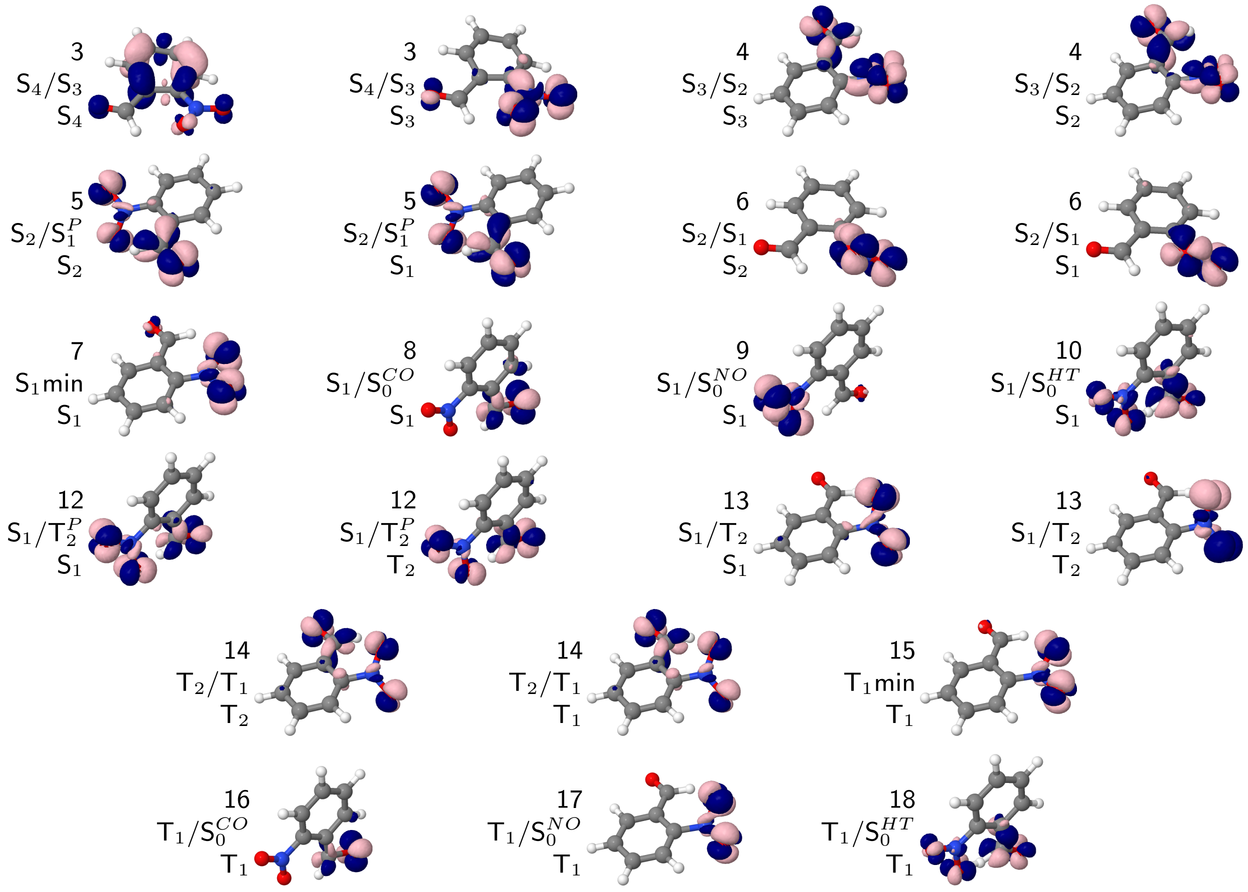

Figure S4: Transition densities between the ground state and the relevant excited states at the different optimized points given in Tables S5 and S6, computed with ADC(2)/cc-pVDZ.

Table S7: MS(12,12)-CASPT2(18,14)/cc-pVTZ excited-state energies[a] at the critical points optimized with MS-CASPT2(12,9)/cc-pVDZ or MS-CASPT2(12,10)/cc-pVDZ (cf. Table S3).

Label	Geom.[b]	Character[c]	S_0	S_1	S_2	S_3	S_4	S_5	T_1	T_2	T_3	SOC[d]
1	S_0min	GS	**0.00**	3.58	3.77	4.20	4.54	**5.58**	3.37	3.49	3.53	
2	S_5/S_4	$\pi\pi^*$ (L_b) and $\pi_{NO2}\pi^*$	1.48	3.83	4.37	4.89	**5.30**	**5.60**	—	—	—	
3	S_4/S_3	$\pi\pi^*$ (L_b) and $n^+_{NO2}\pi^*$	0.57	3.72	4.06	**4.50**	**4.61**	5.46	3.45	3.72	3.78	
4	S_3/S_2	$n_{CO}\pi^*$ and $n^+_{NO2}\pi^*$	0.68	3.78	**4.12**	**4.15**	5.09	6.38	3.41	3.53	3.90	
5	S_2/S_1^P	$n^-_{NO2}\pi^*$ and $n_{CO}\pi^*$	0.60	**3.57**	**3.63**	4.57	4.90	5.84	3.33	3.43	3.44	
6	S_2/S_1	(not comp'd with MS-CASPT2)	—	—	—	—	—	—	—	—	—	
7	S_1min	$n^-_{NO2}\pi^*$	1.38	**2.84**	4.63	4.85	5.31	5.52	2.74	3.35	4.45	
8	S_1/S_0^{CO}	$n_{CO}\pi^*$ and GS	**4.86**	**5.03**	7.69	7.96	8.13	8.18	4.94	6.09	7.71	
9	S_1/S_0^{NO}	$n^-_{NO2}\pi^*$ and GS	**3.10**	**3.15**	5.59	5.73	5.87	5.97	3.01	4.37	5.65	
10	S_1/S_0^{HT}	$(n_{CO}+\sigma_{CH})\pi^*$ and GS	**2.29**	**2.30**	4.08	4.10	5.46	5.62	2.23	3.87	5.15	
11	S_0^{bir}min	$\sigma_{CH}\pi^*_{NO2}$	**1.99**	3.35	4.03	4.95	5.23	5.52	2.05	4.07	4.83	
12	S_1/T_2^P	(not comp'd with MS-CASPT2)	—	—	—	—	—	—	—	—	—	
13	S_1/T_2	$^1n^-_{NO2}\pi^*$ and $^3\pi_{NO2}\pi^*$	1.15	**3.21**	4.64	4.94	5.31	5.61	3.04	**3.20**	4.32	70
14	T_2/T_1	$n^-_{NO2}\pi^*$ and $\pi_{NO2}\pi^*$	0.82	3.23	4.34	4.65	5.15	5.58	**3.11**	**3.17**	4.09	
15	T_1min	$n^-_{NO2}\pi^*$	1.01	2.85	4.41	4.79	5.24	5.40	**2.67**	3.27	4.07	
16	T_1/S_0^{CO}	$^3(n_{CO}+\sigma_{ring\text{-}H})\pi^*$ and GS	**4.54**	4.92	6.94	7.47	7.50	7.61	**4.58**	6.82	7.33	24
17	T_1/S_0^{NO}	$^3n^-_{NO2}\pi^*$ and GS	**3.18**	3.23	5.27	5.90	6.10	6.22	**3.11**	4.19	5.36	64
18	T_1/S_0^{HT}	$^3(n_{CO}+\sigma_{CH})\pi^*$ and GS	**2.07**	2.21	3.90	4.34	5.34	5.67	**2.18**	3.79	5.18	35
19	T_1^{bir}min	$\sigma_{CH}\pi^*_{NO2}$	1.99	3.43	4.24	4.43	5.07	5.43	**1.84**	3.98	4.25	0.5

[a] All energies are in eV relative to the S_0 MS-CASPT2 energy at the S_0min geometry. [b] See Table S3 for optimization settings. [c] According to transition densities. [d] In cm^{-1}.

Table S8: Geometry parameters (bond lengths r, bond angles a, and dihedrals d) and SOCs for the excited-state minima and crossing points optimized at MS-CASPT2/cc-pVDZ level of theory. The numbering of the atoms are introduced in Fig.2

Label	Geom.	$r_{8,12}$ (Å)	$r_{9,13}$ (Å)	$r_{10,12}$ (Å)	$r_{10,11}$ (Å)	$r_{7,5}$ (Å)	$r_{6,10}$ (Å)	$a_{12,10,6}$ (°)	$a_{11,10,6}$ (°)	$a_{8,7,9}$ (°)	$d_{6,5,7,8}$ (°)	$d_{12,10,6,5}$ (°)	$p_{5,7,8,9}$ (°)
1	S_0min	2.29	2.42	1.11	1.22	1.48	1.51	117.0	121.5	125.8	**-35**	**-8**	1
2	S_5/S_4	2.05	2.41	1.13	1.22	1.35	1.41	119.9	123.4	118.9	-12	3	-2
3	S_4/S_3	2.24	2.35	1.11	1.24	1.43	1.48	116.9	120.4	124.7	-8	-4	1
4	S_3/S_2	2.31	2.44	1.11	1.25	1.45	1.48	118.4	121.5	127.9	-44	-6	14
5	S_2/S_1^P	2.35	2.42	1.10	1.30	1.43	1.45	121.2	122.1	119.1	-35	-2	1
6	S_2/S_1	—	—	—	—	—	—	—	—	—	—	—	—
7	S_1min	2.12	2.41	1.11	1.22	**1.38**	1.50	116.7	121.7	**107.0**	0	-1	0
8	S_1/S_0^{CO}	2.60	**1.48**	1.08	**1.57**	1.47	**1.35**	**141.8**	**103.2**	125.6	0	-10	0
9	S_1/S_0^{NO}	2.17	2.45	1.11	1.22	**1.33**	1.49	116.5	122.3	**92.1**	0	0	0
10	S_1/S_0^{HT}	**1.20**	2.72	**1.36**	1.21	1.41	1.47	**99.5**	**129.5**	118.5	0	0	0
11	S_0^{bir}min	**0.99**	2.60	**1.96**	1.20	1.41	1.47	**93.2**	**129.3**	117.5	-12	0	4
12	S_1/T_2^P	—	—	—	—	—	—	—	—	—	—	—	—
13	S_1/T_2	2.17	2.40	1.10	1.23	1.40	1.50	116.8	121.0	113.0	2	0	**12**
14	T_2/T_1	2.11	2.40	1.11	1.23	1.40	1.49	117.4	121.6	110.8	5	-1	0
15	T_1min	2.12	2.41	1.11	1.22	**1.38**	1.50	116.7	121.7	**107.0**	0	-1	0
16	T_1/S_0^{CO}	2.83	**1.12**	1.08	**1.43**	1.46	**1.40**	**136.7**	**106.6**	124.9	12	13	-16
17	T_1/S_0^{NO}	2.18	2.40	1.11	1.23	1.39	1.50	116.5	120.9	**112.0**	1	0	13
18	T_1/S_0^{HT}	**1.23**	2.75	**1.33**	1.21	1.41	1.45	**100.0**	**131.1**	118.4	0	0	0
19	T_1^{bir}min	**1.00**	2.59	**1.78**	1.20	1.40	1.48	**92.5**	**128.8**	115.7	-3	0	1

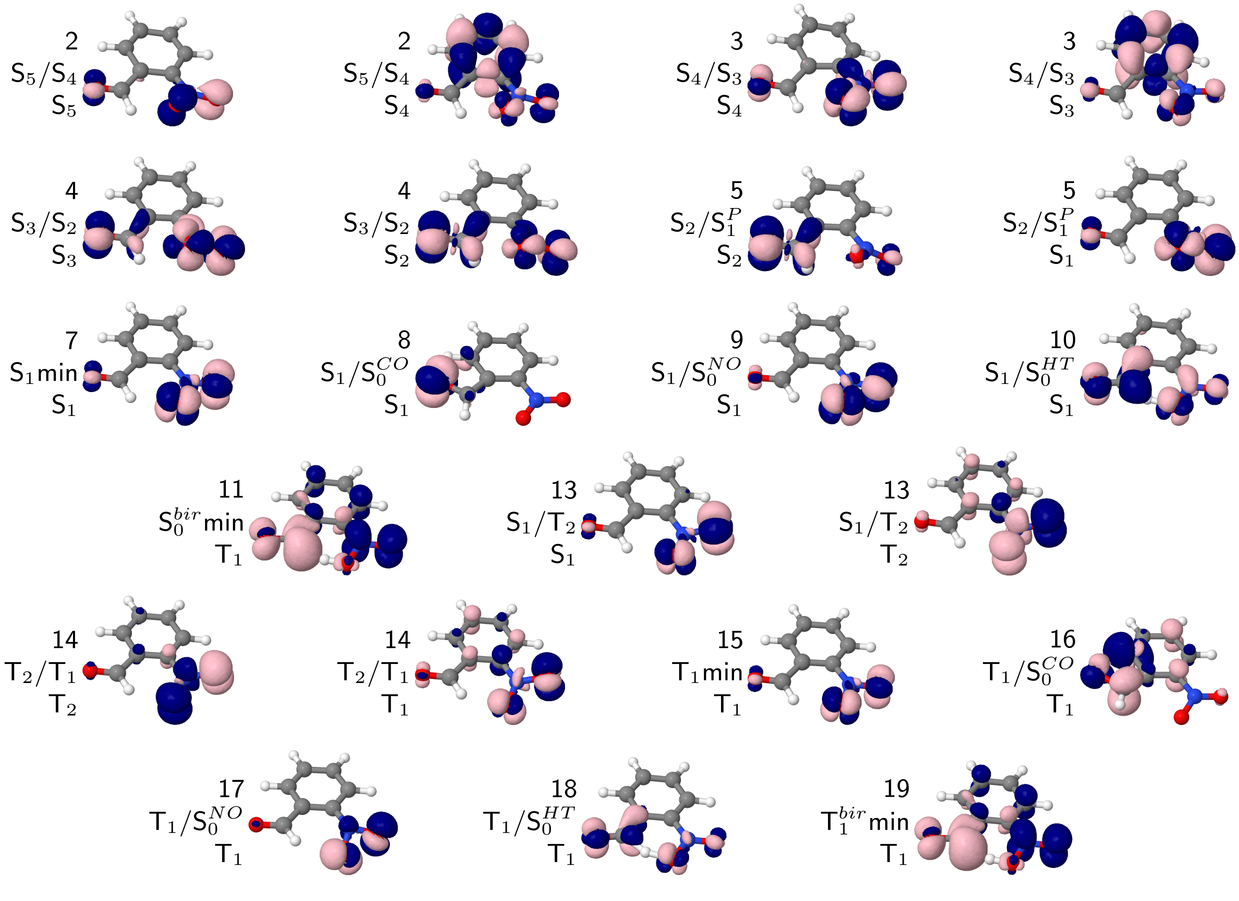

Figure S5: Transition densities between the ground state and the relevant excited states at the different optimized points given in Tables S7 and S8, computed with MS-CASPT2(18,14)/cc-pVDZ.

Table S9: Spin-orbit couplings at ADC(2)/cc-pVDZ level of theory at geometry 13 (S_1/T_2) in cm^{-1}.

	$^3n_{CO}\pi^*$	$^3n^-_{NO2}\pi^*$	$^3n^+_{NO2}\pi^*$	$^3\pi_{NO2}\pi^*$	$^3\pi\pi^*$ (L_a)
$^1n_{CO}\pi^*$	1	2	1	1	15
$^1n^-_{NO2}\pi^*$	3	10	30	50	7
$^1n^+_{NO2}\pi^*$	2	32	3	16	10
$^1\pi_{NO2}\pi^*$	2	39	17	8	4
$^1\pi\pi^*$ (L_b)	4	6	4	2	1

Table S10: Spin-orbit couplings at MS-CASPT2(18,14)/cc-pVDZ level of theory at geometry 13 (S_1/T_2) in cm^{-1}.

	$^3n_{CO}\pi^*$	$^3n^-_{NO2}\pi^*$	$^3n^+_{NO2}\pi^*$	$^3\pi_{NO2}\pi^*$	$^3\pi\pi^*$ (L_a)
$^1n_{CO}\pi^*$	0	4	19	11	15
$^1n^-_{NO2}\pi^*$	4	3	9	70	12
$^1n^+_{NO2}\pi^*$	2	12	10	42	12
$^1\pi_{NO2}\pi^*$	8	25	4	4	7
$^1\pi\pi^*$ (L_b)	5	6	2	6	3

S4 Excited-State Potential Energy Surfaces

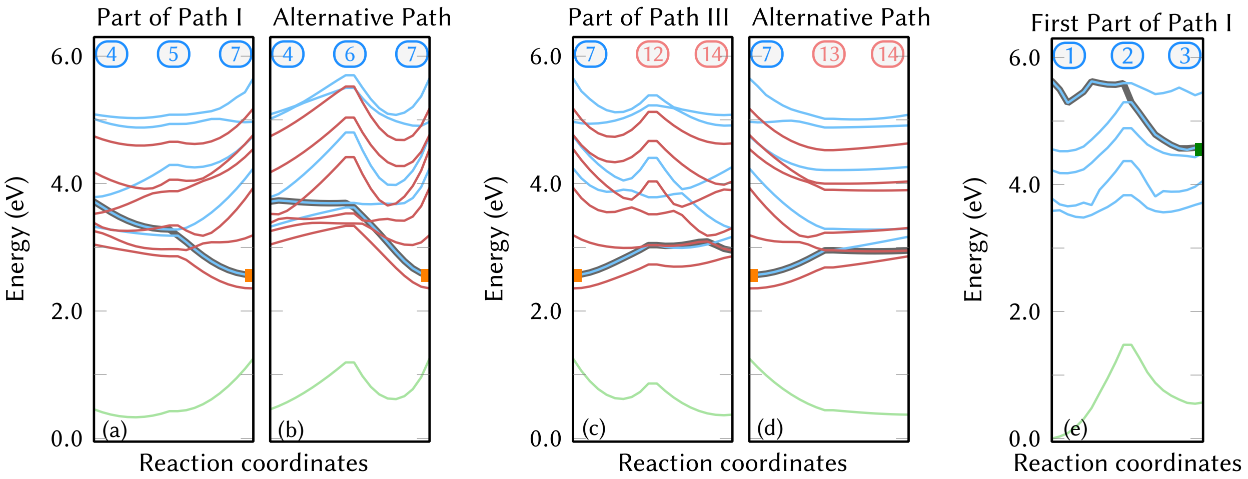

Figure S6: LIIC scans (a)–(d) at ADC(2)/cc-pVDZ level of theory for the relaxations: $S_3/S_2 \rightarrow S_2/S_2 \rightarrow S_1$min (orange rectangle) and S_1min (purple rectangle) $\rightarrow S_1/T_2 \rightarrow T_2/T_1$ (pink rectangle) and (e) at MS(12)-CASPT2(18,14)/cc-pVTZ level pf theory for the relaxation: S_5FC $\rightarrow S_5/S_4 \rightarrow S_4/S_3$ (green rectangle). The labels of the crossing points and minima are given in Table S5.

In Figure S6, we show potential energy scans (ADC(2)/cc-pVDZ level of theory) of alternative pathway segments (see Figure 3 for the majority of scans). Panels a and b compare the relaxation pathways from S_3/S_2 to S_1min through two different S_2/S_1 MECIs. The two MECIs—S_2/S_1^P (5) and S_2/S_1 (6)—differ most prominently in a planarized or pyramidalized nitro group and the C-N and C=O bond lengths (Table S6). The planarized MECI S_2/S_1^P (5) exhibits a lower energy (Table S5) and thus likely provides the dominant pathway.

Figure S6c and d compares two alternative pathways for ISC through two different S_1/T_2 MECPs. These MECPs show similar geometries as the two S_2/S_1 MECIs, with one MECP having a rather planar nitro group and the other MECP having a pyramidalized nitro group (Table S6). The planarized MECP S_1/T_2^P (12) shows slightly lower energies (Table S5), whereas the pyramidalized MECP S_1/T_2 (13) exhibits much larger SOC matrix elements (Table S6). As both pathways are hampered by small SOC and/or high energy barrier, ISC from the S_1 minimum is not expected to be very efficient.

Figure S6e shows the first part of the LIIC scan from the FC point to the S_5/S_4 MECI and then to the S_4/S_3 MECI. it shows that the S_5/S_4 MECI was not optimized well with MS(12)-CASPT2(12,9). However, we assume that there is an easily accessible CI from the S_5 to the S_4.

S5 Dynamics

The most relevant/coherent geometry parameters are shown in Figure 7 in the main text. In Figure S7, we show the time evolution of further parameters to show that they do not exhibit coherent vibrations.

Figure S7a shows the C=O bond length. According to the optimizations of critical points, this coordinate gives access to the S_1/S_0^{CO} and T_1/S_0^{CO} crossing points. However, this bond length does not vary much among the remaining critical points. Consequently, the dynamics shows a relatively constant bond length of about 1.25 Å with no coherent oscillations. Bond lengths above 1.4 Å are rarely visited (but see the discussion in Section "Decay to the ground state" in the main text).

Figure S7b presents the C–H bond length of the aldehyde group. Even though the optimized structures exhibit nearly constant values for this parameter, excitation appears to induce weak but coherent oscillations (period 11–12 fs). Later, the average seems to increase slightly, as some trajectories attempt HT (see Section "Decay to the ground state" in the main text).

In Figure S7c, the distribution of all six intra-ring C–C–C bond angles is shown. All six angles are distributed between 115° and 125° at all times, indicating that the ring is not deformed in-plane. The fact that the average of all six angles remains at precisely 120° further indicates that the ring remains planar over the simulated time frame. The latter observation is supported by the plot of the ring puckering amplitude (i.e., the average displacement of the ring atoms from the mean plane) in panel d.

Panels e–h present the pyramidalization angles of the two aromatic C atoms carrying the two functional groups as well as the pyramidalization angles of the two functional groups. Due to the symmetry of the pyramidalization angles around 0°, all averages tend to be close to zero. However, it can be observed that all four distributions become broader in time, indicating that the molecule becomes less rigid in the excited state. In particular, among the four pyramidalization angles, the one of the NO_2 group has the most narrow distribution in the beginning (standard deviation of 6°) and the broadest one (15°) in the end.

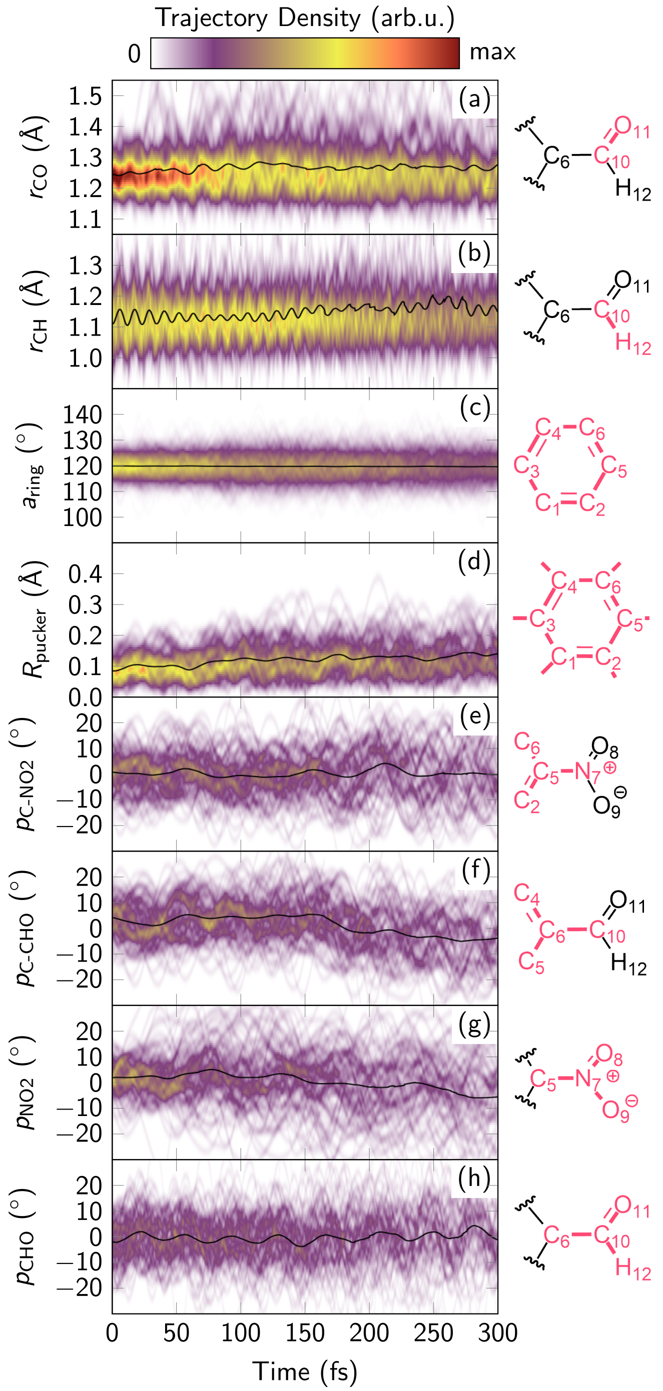

Figure S7: Time evolution of nuclear geometry parameters. Black lines indicate the average. (a) C–O distance. (b) $C–H_{aldehyde}$ distance. (c) Average intra-ring angle. (d) Ring puckering amplitude. (e) Pyramidalization of aromatic C carrying NO_2 group. (f) Pyramidalization of aromatic C carrying CHO group. (g) Pyramidalization of N atom. (h) Pyramidalization of aldehyde C atom.

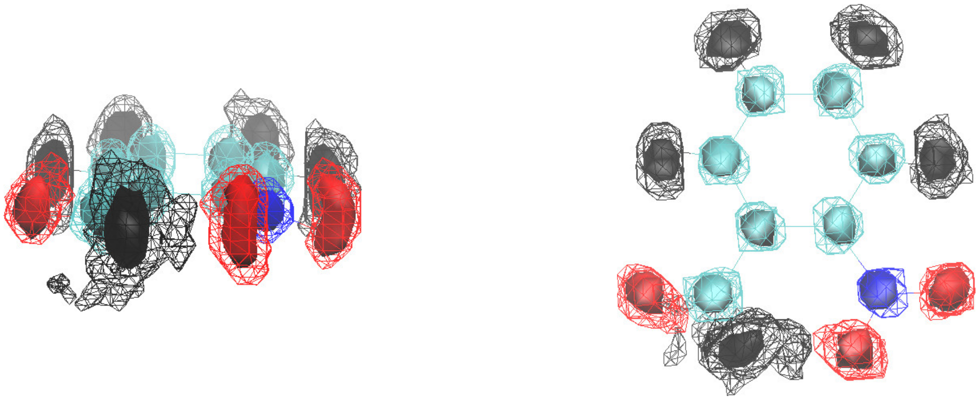

Figure S8: Distribution of atomic positions from all 96 trajectories, showing that oNBA is rather rigid in the excited state.

Figure S8 shows a three-dimensional histogram (plotted as isosurfaces) of the distribution of atomic positions in space. The figure shows how mobile the different atoms are. For oNBA, we observe a very rigid ring and only limited motion at the functional groups. The most mobile atoms are the aldehyde H atom—which moves out-of-plane to some extent and transfers to the nitro group for some trajectories—and the nitro group O atoms—which move out-of-plane during the torsional motion of the nitro group.

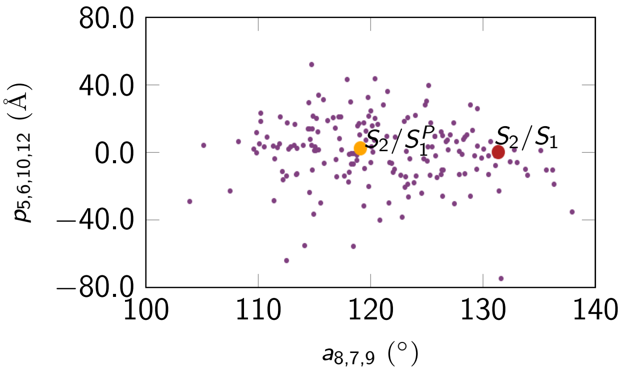

Figure S9: Scatter plot between two geometrical parameter: bond angle formed by O_8-N_7-O_9 and the pyramidalization angle C_5-C_6-C_{10}-C_{12}. The purple circles presents the geometrical parameters obtained from the trajectories that undergo transition from S_2 to the S_1 state. The bigger circles demonstrate the optimized geometries S_2/S_1^P (orange, geometry 5) and S_2/S_1 (red, geometry 6) crossing points.

Figure S9 shows the distribution of bond angle O_8–N_7–O_9 and pyramidalization angle C_5–C_6–C_{10}–C_{12}. The orange and red dots correspond to the S_2/S_1^P and S_2/S_1 geometries (geometries 5 and 6, respectively). The figure shows that the planarized S_2/S_1^P conical intersection is preferred. This is consistent with the lower energy of this conical intersection compared to S_2/S_1 and indicates that the $n_{CO}\pi^*$ state is visited on the way to the S_1 minimum (see characters of the S_1 and S_2 states at the S_2/S_1^P conical intersection in Table S5).

Figure S10 compares the adiabatic energies and energy gaps of the obtained surface hops with the optimized MECIs/MECPs. We only show hops $S_4 \rightarrow S_3$, $S_3 \rightarrow S_2$, $S_2 \rightarrow S_1$, $S_1 \rightarrow T_2$, and $T_2 \rightarrow T_1$ because for those state pairs we have optimized crossing points.

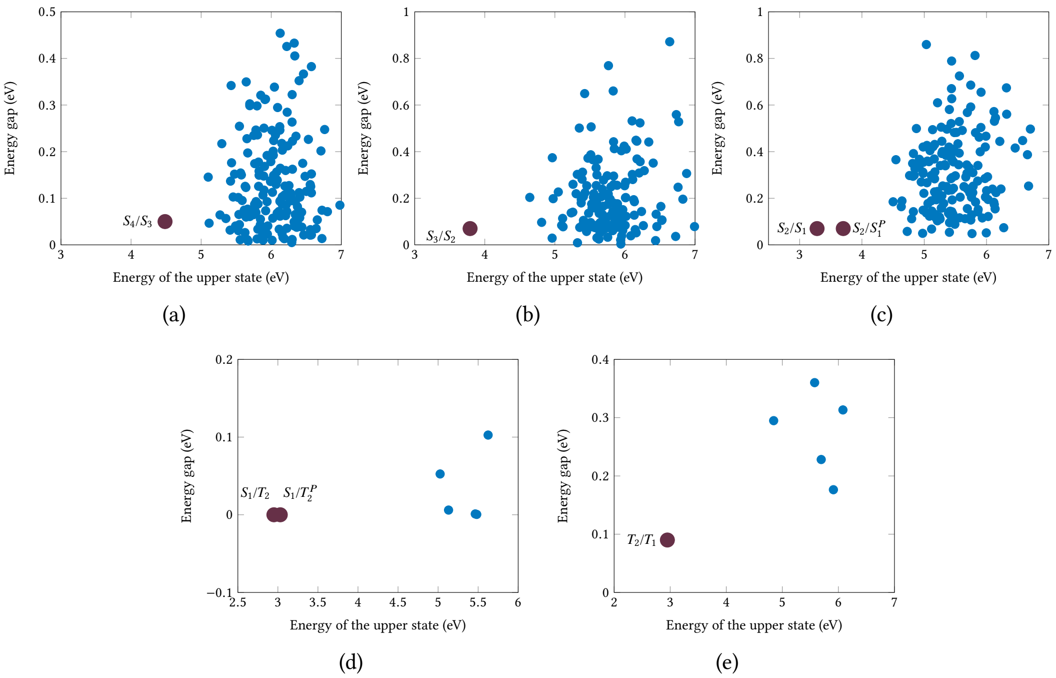

Figure S10: Scatter plots showing the adiabatic energies and energy gaps at the obtained hopping events from 96 trajectories. The larger dark dots show the location of the optimized MECI/MECP for comparison.

Figure S11 furthermore shows the distribution of the most relevant geometric parameters for the majority of the surface hopping events (for the same state pairs). The information in both figures indicates that surface hops do not occur directly at the optimized MECIs/MECPs, but rather distributed relatively widely around them.

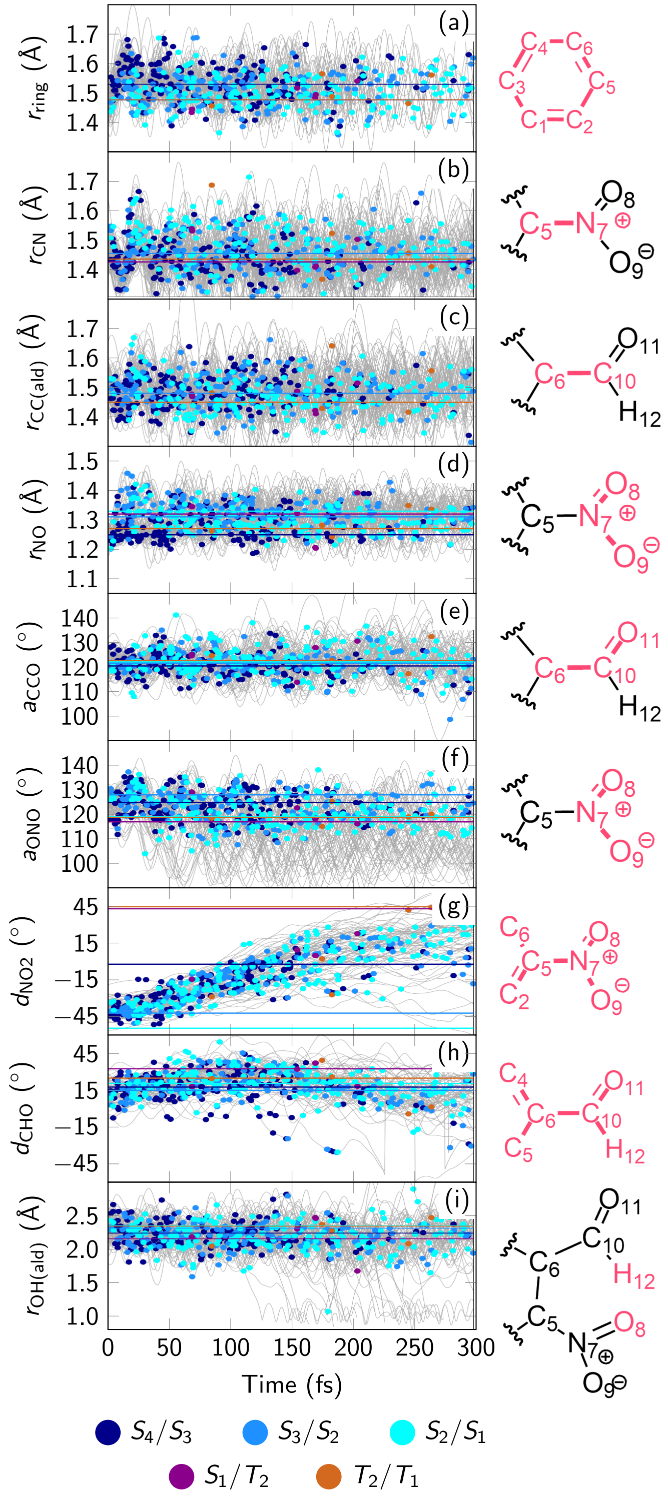

Figure S11: Line plots of the evolution of the geometry parameters in Figure 8 and scatter plots of the time and positions of the observed surface hops. Different colors indicate different pairs of state. Horizontal lines indicate the parameters of the optimized MECIs/MECPs.

S6 Coordinates

Equilibrium geometry optimized with MP2/cc-pVDZ.

```
16
S0 (1)
C -0.010226 +0.193098 +0.014007
C +0.189652 +0.361402 +1.391667
C +1.057213 -0.213141 -0.808246
C +2.320977 -0.441201 -0.249556
C +1.456795 +0.105039 +1.932928
C +2.549711 -0.291217 +1.135153
N +1.587483 +0.235807 +3.397079
O +2.338096 -0.560729 +3.969853
O +0.918053 +1.115660 +3.943651
C +3.960407 -0.497303 +1.621425
O +4.828491 -0.951127 +0.889365
H +4.181797 -0.194547 +2.664454
H +3.172547 -0.738149 -0.869147
H +0.902567 -0.344039 -1.883889
H -1.000123 +0.377730 -0.415018
H -0.617506 +0.680286 +2.055392
```

The following coordinates were optimized at ADC(2)/cc-pVDZ level of theory.

```
16
S4/S3 (3)
C +0.023571 +0.302515 -0.021479
C +0.252643 +0.507586 +1.366303
C +1.038768 -0.277647 -0.829129
C +2.274318 -0.590005 -0.216417
C +1.457073 +0.029562 +2.022935
C +2.578178 -0.291000 +1.190631
N +1.503200 +0.090273 +3.445846
O +2.539785 -0.300952 +4.010675
O +0.489211 +0.511419 +4.036475
C +4.006632 -0.378957 +1.565250
O +4.850644 -0.745181 +0.737315
H +4.269138 -0.103262 +2.602813
H +3.085149 -1.067905 -0.777270
H +0.869523 -0.499856 -1.885753
H -0.936795 +0.603920 -0.450634
H -0.465104 +1.047057 +1.991557
```

```
16
S3/S2 (4)
C +0.011216 +0.032671 +0.005141
C -1.180057 +0.014765 +0.745715
C -0.028498 +0.039678 -1.402961
C -1.259616 +0.012212 -2.066548
C -2.408026 +0.007822 +0.066138
C -2.484142 -0.012950 -1.353863
N -3.624803 +0.023083 +0.859497
O -4.553558 +0.840945 +0.421660
O -3.549400 -0.551197 +2.029127
C -3.738115 -0.131797 -2.129089
O -3.743405 -0.067196 -3.376114
H -4.682656 -0.304444 -1.579490
H -1.311459 -0.004409 -3.159137
H +0.902041 +0.056539 -1.978873
H +0.970608 +0.041758 +0.532144
H -1.177930 +0.014522 +1.837953
```

```
16
S2/S1P (5)
C -0.007574 +0.024103 +0.001266
C +1.197620 +0.004697 -0.716351
C +0.016472 +0.048942 +1.412396
C +1.234157 +0.030958 +2.095894
C +2.416800 +0.007879 -0.024535
C +2.476801 -0.015698 +1.399913
N +3.616156 +0.052280 -0.805238
O +4.635458 +0.688281 -0.420801
O +3.645302 -0.550912 -1.946869
C +3.683271 -0.163230 +2.185559
O +3.666071 -0.158997 +3.482139
H +4.662940 -0.309361 +1.703885
H +1.267580 +0.021330 +3.190650
H -0.921600 +0.071367 +1.976354
H -0.959809 +0.032441 -0.537921
H +1.218676 +0.010683 -1.810123
```

```
16
S2/S1 (6)
C -0.009917 +0.157930 +0.042353
C +0.206043 +0.357379 +1.414760
C +1.049589 -0.234828 -0.798419
C +2.325911 -0.431159 -0.260174
C +1.489176 +0.136219 +1.926085
C +2.569804 -0.258088 +1.119807
N +1.741671 +0.334825 +3.392232
O +2.106561 -0.831525 +3.901389
O +0.938731 +1.290644 +3.863741
C +3.966485 -0.468957 +1.625994
O +4.881132 -0.803607 +0.886417
H +4.124130 -0.302265 +2.712316
H +3.173576 -0.733565 -0.883097
H +0.876089 -0.379456 -1.869169
H -1.010453 +0.322986 -0.370477
H -0.592595 +0.681036 +2.085360
```

```
16
S1min (7)
C +0.013333 -0.008696 +0.012612
C -1.194613 -0.001002 +0.708074
C +0.033257 -0.002781 -1.401349
C -1.173231 +0.010838 -2.106979
C -2.413286 +0.012882 -0.024239
C -2.424279 +0.019038 -1.451216
N -3.568271 +0.019909 +0.729175
O -4.820709 +0.033628 +0.332951
O -3.633047 +0.014313 +2.046887
C -3.652550 +0.033304 -2.301146
O -3.605508 +0.037868 -3.525975
H -4.625532 +0.039493 -1.762031
H -1.192324 +0.015667 -3.201322
H +0.986749 -0.008845 -1.938314
H +0.952457 -0.019376 +0.575423
H -1.231054 -0.005313 +1.800589
```

```
16
S1/S0CO (8)
C -1.217246 -2.232079 +0.072677
C +0.111472 -1.823342 +0.056779
C -2.271028 -1.284346 +0.053787
C -1.971949 +0.081966 +0.019824
C +0.402323 -0.422668 +0.023894
C -0.644955 +0.557644 +0.001493
N +1.706650 -0.088578 -0.001591
O +2.392571 +1.050089 -0.000063
O +2.807943 -0.863920 +0.031597
C -0.418733 +2.030719 -0.035849
O -1.338200 +2.841495 -0.049011
H +0.642037 +2.366913 -0.052793
H -2.764314 +0.837258 +0.004902
H -3.312209 -1.621063 +0.066478
H -1.444298 -3.302986 +0.100374
H +0.939924 -2.537572 +0.072709
```

```
16
S1/S0NO (9)
C -1.148088 -2.271167 -0.112725
C +0.187914 -1.844528 -0.075828
C -2.196868 -1.317736 -0.114158
C -1.908147 +0.038009 -0.079328
C +0.496799 -0.481150 -0.040202
C -0.543479 +0.520330 -0.040901
N +1.907730 -0.098295 -0.002733
O +2.165723 +1.115161 +0.026879
O +2.756946 -0.995434 -0.002595
C -0.383057 +1.882687 -0.009280
O -1.734418 +2.764978 -0.019105
H +0.450793 +2.581749 +0.022653
H -2.714157 +0.782358 -0.080230
H -3.239582 -1.650512 -0.142879
H -1.373748 -3.341886 -0.140297
H +1.014827 -2.558506 -0.073794
```

```
16
S1/S0HT (10)
C -1.163579 -2.296132 -0.064804
C +0.160885 -1.854432 -0.069660
C -2.254721 -1.391965 -0.006180
C -1.997467 -0.023187 +0.048496
C +0.417925 -0.462984 -0.012976
C -0.664397 +0.459294 +0.045893
N +1.760294 -0.039493 -0.019661
O +2.035099 +1.269919 +0.033554
O +2.718328 -0.840458 -0.072773
C -0.366398 +1.882864 +0.102049
O -1.103184 +2.850288 +0.156657
H +0.949768 +1.863790 +0.078574
H -2.807576 +0.712927 +0.094219
H -3.283048 -1.766109 -0.003684
H -1.358065 -3.373288 -0.108330
H +1.004798 -2.545351 -0.115407
```

```
16
S1/T2P (12)
C -1.118953 -2.228833 +0.124887
C +0.195981 -1.755451 +0.116120
C -2.197827 -1.316964 +0.070793
C -1.948986 +0.052217 -0.001943
C +0.445558 -0.369694 +0.050051
C -0.622943 +0.576233 -0.024164
N +1.812952 +0.027605 +0.068091
O +2.178029 +1.212217 +0.370284
O +2.739458 -0.809714 -0.212639
C -0.472591 +2.012801 -0.164737
O -1.474000 +2.807484 -0.239828
H +0.533839 +2.460631 -0.222233
H -2.773686 +0.770225 -0.057919
H -3.229274 -1.683400 +0.082711
H -1.305226 -3.305705 +0.184175
H +1.050481 -2.435179 +0.169527
```

```
16
S1/T2 (13)
C -0.001456 -0.024557 +0.010216
C -1.223398 +0.029697 +0.698468
C +0.024115 -0.062079 -1.396454
C -1.182235 -0.024691 -2.104467
C -2.424084 +0.054527 -0.024566
C -2.422541 +0.047867 -1.434128
N -3.693196 +0.068209 +0.705474
O -4.532912 -0.888037 +0.318274
O -3.651954 +0.430408 +1.966315
C -3.657583 +0.185852 -2.269399
O -3.656683 +0.009698 -3.480520
H -4.580303 +0.502518 -1.738117
H -1.202556 -0.032091 -3.198703
H +0.977842 -0.111609 -1.930678
H +0.933262 -0.048563 +0.579946
H -1.254926 +0.053780 +1.791480
```

```
16
T2/T1 (14)
C +0.016849 -0.022676 +0.015103
C -1.199818 -0.006427 +0.708870
C +0.016790 -0.042125 -1.396680
C -1.188608 -0.024719 -2.102950
C -2.407943 -0.008772 -0.007094
C -2.441865 +0.018160 -1.431241
N -3.616782 -0.045350 +0.755848
O -4.625858 -0.709504 +0.361432
O -3.692359 +0.583849 +1.866897
C -3.648606 +0.153135 -2.231580
O -3.613136 +0.124160 -3.518110
H -4.633371 +0.283849 -1.758079
H -1.197443 -0.009421 -3.197673
H +0.964707 -0.059054 -1.944109
H +0.959684 -0.030876 +0.570416
H -1.240850 -0.013301 +1.802090
```

```
16
T1min (15)
C +0.012870 -0.009289 +0.007740
C -1.201140 +0.022243 +0.701683
C +0.034629 -0.028163 -1.402373
C -1.171632 -0.011586 -2.110107
C -2.408368 +0.023153 -0.030844
C -2.419052 +0.030969 -1.449766
N -3.595844 +0.107474 +0.718801
O -4.786225 -0.309098 +0.359454
O -3.636699 +0.106359 +2.036806
C -3.653588 +0.092551 -2.293998
O -3.612758 +0.054364 -3.517384
H -4.618983 +0.189480 -1.751393
H -1.191080 -0.018007 -3.204367
H +0.988347 -0.055975 -1.938226
H +0.950416 -0.024694 +0.573045
H -1.239501 +0.021146 +1.794070
```

```
16
T1/S0CO (16)
C -1.148649 -2.275624 -0.112542
C +0.185053 -1.847864 -0.075744
C -2.194879 -1.319189 -0.114251
C -1.906148 +0.036866 -0.079735
C +0.493546 -0.482539 -0.040618
C -0.543228 +0.519046 -0.041180
N +1.901708 -0.096731 -0.003054
O +2.155551 +1.118322 +0.027761
O +2.755325 -0.989936 -0.003825
C -0.371233 +1.882405 -0.008887
O -1.716659 +2.763775 -0.018338
H +0.447975 +2.597781 +0.023185
H -2.718443 +0.774775 -0.081169
H -3.238531 -1.649460 -0.142949
H -1.376017 -3.345787 -0.139762
H +1.013816 -2.559779 -0.073411
```

```
16
T1/S0NO (17)
C +0.007946 -0.041184 +0.002095
C -1.212556 +0.025707 +0.691888
C +0.034339 -0.059264 -1.405649
C -1.166712 -0.008623 -2.125670
C -2.402369 +0.058755 -0.050891
C -2.407857 +0.069211 -1.462648
N -3.642513 +0.189749 +0.665386
O -4.664632 -0.628275 +0.469988
O -3.668210 +0.196199 +2.000420
C -3.658986 +0.165675 -2.278975
O -3.648001 +0.149475 -3.502217
H -4.610316 +0.259971 -1.708984
H -1.178952 -0.013406 -3.220007
H +0.990506 -0.112972 -1.935354
H +0.942979 -0.083734 +0.570091
H -1.263274 +0.023643 +1.783668
```

```
16
T1/S0HT (18)
C -1.186506 -2.283262 +0.005883
C +0.134800 -1.844410 -0.030478
C -2.276812 -1.371426 +0.059335
C -2.021490 -0.005343 +0.075700
C +0.395328 -0.449714 -0.013781
C -0.684361 +0.479433 +0.039345
N +1.734858 -0.030575 -0.050409
O +2.010106 +1.273462 -0.036901
O +2.693580 -0.832009 -0.095691
C -0.385384 +1.895872 +0.055207
O -1.105922 +2.878693 +0.097346
H +0.908388 +1.886638 +0.011630
H -2.832083 +0.730478 +0.116447
H -3.305289 -1.744486 +0.086918
H -1.385276 -3.360307 -0.006870
H +0.978106 -2.536424 -0.071368
```

Hereafter, the coordinates are shown which were optimized at MS-CASPT2/cc-pVDZ level of theory with two different active spaces.

```
16
S5/S4 (2)
C -1.713954 +0.308690 -1.167037
C -1.528808 +0.477382 +0.191822
C -0.667048 -0.185259 -2.048942
C +0.560460 -0.440586 -1.466917
C -0.282865 +0.226637 +0.896184
C +0.816639 -0.255090 +0.020340
N -0.172038 +0.305127 +2.235053
O +0.853295 -0.272654 +2.799535
O -1.004517 +0.965179 +2.975972
C +2.152722 -0.421205 +0.428332
O +3.051578 -0.760077 -0.331242
H +2.441743 -0.222978 +1.503222
H +1.403717 -0.763096 -2.091756
H -0.813730 -0.325575 -3.127801
H -2.704139 +0.532481 -1.587668
H -2.393055 +0.831021 +0.770905
```

```
16
S1min (7)
C -1.741397 +0.294003 -1.167964
C -1.549735 +0.446743 +0.209564
C -0.678904 -0.117266 -1.994053
C +0.583170 -0.373688 -1.429333
C -0.272436 +0.187858 +0.763978
C +0.822137 -0.230851 -0.056811
N -0.153898 +0.357865 +2.132703
O +0.882425 +0.188352 +2.870180
O -1.104552 +0.739227 +2.905045
C +2.213825 -0.527496 +0.430052
O +3.097375 -0.893988 -0.331648
H +2.400664 -0.395669 +1.517042
H +1.425137 -0.694137 -2.049210
H -0.831821 -0.237642 -3.069309
H -2.727414 +0.495881 -1.595636
H -2.364577 +0.760807 +0.865399
```

```
16
S1/S0CO (8)
C -1.663640 +0.285044 -1.195866
C -1.559184 +0.447593 +0.234099
C -0.550442 -0.131438 -1.940873
C +0.648835 -0.384627 -1.266695
C -0.381108 +0.210851 +0.889046
C +0.788604 -0.218069 +0.153423
N -0.274854 +0.402678 +2.341061
O +0.835636 +0.184035 +2.840470
O -1.279195 +0.755799 +2.954793
C +2.074087 -0.504055 +0.446705
O +2.699506 -0.831509 -0.957901
H +2.799547 -0.439495 +1.250555
H +1.523108 -0.777678 -1.857750
H -0.618702 -0.273308 -3.023379
H -2.616586 +0.503166 -1.684120
H -2.425610 +0.771013 +0.816431
```

```
16
S1/S0NO (9)
C -1.752936 +0.286794 -1.177271
C -1.568345 +0.446095 +0.190943
C -0.682099 -0.122003 -2.011827
C +0.586640 -0.370664 -1.448574
C -0.271823 +0.186625 +0.755140
C +0.828885 -0.232226 -0.079300
N -0.145212 +0.350623 +2.072081
O +0.844605 +0.222985 +2.946902
O -0.991261 +0.722649 +3.011208
C +2.202414 -0.523251 +0.432899
O +3.114679 -0.873496 -0.303576
H +2.359100 -0.409303 +1.529475
H +1.429012 -0.686267 -2.071800
H -0.836443 -0.243746 -3.087334
H -2.738216 +0.482743 -1.609956
H -2.378999 +0.762443 +0.850992
```

16
S1/S0HT (10)
C -1.735061 +0.297294 -1.158749
C -1.516676 +0.452163 +0.213549
C -0.696806 -0.123348 -2.024261
C +0.573313 -0.389750 -1.509767
C -0.235606 +0.178613 +0.736155
C +0.819157 -0.246373 -0.126267
N -0.041514 +0.345314 +2.127275
O +1.151010 +0.095593 +2.655475
O -0.947645 +0.718579 +2.872573
C +2.136227 -0.521301 +0.455982
O +3.154948 -0.878851 -0.083738
H +1.867121 -0.247861 +1.759762
H +1.397317 -0.718971 -2.150358
H -0.886770 -0.242380 -3.095354
H -2.730489 +0.505632 -1.563527
H -2.308526 +0.775648 +0.891250

16
S0minBir (11)
C -1.728965 +0.303975 -1.168081
C -1.495954 +0.446217 +0.199406
C -0.704310 -0.137971 -2.035267
C +0.560095 -0.418870 -1.521215
C -0.220728 +0.141806 +0.734006
C +0.825667 -0.286475 -0.131719
N -0.069303 +0.313609 +2.122761
O +1.080764 -0.197050 +2.722820
O -0.921364 +0.803925 +2.855951
C +2.221164 -0.452743 +0.308752
O +3.148539 -0.933288 -0.274661
H +1.822717 +0.097758 +2.144918
H +1.377681 -0.745505 -2.170823
H -0.900070 -0.256333 -3.105074
H -2.721689 +0.535193 -1.566709
H -2.274245 +0.785751 +0.884937

16
S1/T2 (13)
C -1.737237 +0.297373 -1.155495
C -1.517643 +0.485166 +0.219452
C -0.687240 -0.133815 -1.978679
C +0.578731 -0.378704 -1.427155
C -0.245863 +0.214553 +0.765888
C +0.839032 -0.217484 -0.052218
N -0.093624 +0.430054 +2.137151
O +0.908492 +0.350081 +2.870120
O -1.286915 +0.451276 +2.850429
C +2.222903 -0.541460 +0.428373
O +3.091771 -0.917086 -0.354046
H +2.413920 -0.427098 +1.510503
H +1.412055 -0.712519 -2.050538
H -0.852723 -0.269764 -3.050290
H -2.725131 +0.510046 -1.575362
H -2.320528 +0.859380 +0.861865

16
T2/T1 (14)
C -1.740510 +0.294510 -1.165000
C -1.544805 +0.447200 +0.212035
C -0.676189 -0.115227 -1.991043
C +0.583497 -0.370259 -1.429107
C -0.270083 +0.190181 +0.767512
C +0.825141 -0.228980 -0.049591
N -0.143035 +0.366042 +2.147703
O +0.916822 +0.271483 +2.842189
O -1.154945 +0.670935 +2.892711
C +2.203267 -0.536837 +0.434741
O +3.089482 -0.909602 -0.334351
H +2.401837 -0.417705 +1.517511
H +1.423129 -0.690998 -2.052013
H -0.827800 -0.234966 -3.067129
H -2.726490 +0.498012 -1.592258
H -2.359318 +0.766210 +0.866091

16
T1min (15)
C -1.741397 +0.294003 -1.167964
C -1.549735 +0.446743 +0.209564
C -0.678904 -0.117266 -1.994053
C +0.583170 -0.373688 -1.429333
C -0.272436 +0.187858 +0.763978
C +0.822137 -0.230851 -0.056811
N -0.153898 +0.357865 +2.132703
O +0.882425 +0.188352 +2.870180
O -1.104552 +0.739227 +2.905045
C +2.213825 -0.527496 +0.430052
O +3.097375 -0.893988 -0.331648
H +2.400664 -0.395669 +1.517042
H +1.425137 -0.694137 -2.049210
H -0.831821 -0.237642 -3.069309
H -2.727414 +0.495881 -1.595636
H -2.364577 +0.760807 +0.865399

16
T1/S0CO (16)
C -1.700009 +0.258290 -1.192130
C -1.589202 +0.392025 +0.239817
C -0.554416 -0.080101 -1.974582
C +0.586625 -0.344677 -1.216279
C -0.373858 +0.221115 +0.904869
C +0.774298 -0.210771 +0.198360
N -0.277239 +0.438940 +2.342812
O +0.868496 +0.456462 +2.798881
O -1.266810 +0.884044 +2.913223
C +2.120107 -0.555054 +0.330639
O +2.558371 -0.990548 -0.959860
H +2.795681 -0.782189 +1.137762
H +1.818853 -0.498504 -1.636463
H -0.619350 -0.211823 -3.058416
H -2.678295 +0.364809 -1.670937
H -2.463252 +0.657982 +0.842306

16
T1/S0NO (17)
C -1.744014 +0.281430 -1.171758
C -1.553914 +0.384302 +0.215038
C -0.674808 -0.076727 -2.012159
C +0.592964 -0.338296 -1.456723
C -0.275621 +0.140596 +0.744683
C +0.808244 -0.258646 -0.072779
N -0.133743 +0.173265 +2.154723
O +0.908458 +0.664128 +2.792596
O -1.000768 +0.748233 +2.947218
C +2.167852 -0.604939 +0.458693
O +3.106063 -0.871801 -0.277322
H +2.278300 -0.627558 +1.566082
H +1.441298 -0.627249 -2.084089
H -0.823559 -0.152207 -3.093105
H -2.731745 +0.485593 -1.594828
H -2.365007 +0.679876 +0.883729

16
T1/S0HT (18)
C -1.738462 +0.292558 -1.156907
C -1.522385 +0.441066 +0.212397
C -0.691843 -0.119512 -2.024208
C +0.574450 -0.382455 -1.511416
C -0.236222 +0.174852 +0.738546
C +0.828176 -0.240374 -0.118837
N -0.059264 +0.337272 +2.129080
O +1.143083 +0.095042 +2.656946
O -0.961951 +0.691875 +2.875417
C +2.135834 -0.505717 +0.452830
O +3.173719 -0.861015 -0.062433
H +1.885487 -0.247272 +1.732513
H +1.397941 -0.701274 -2.157665
H -0.881107 -0.230433 -3.096319
H -2.733280 +0.498607 -1.563978
H -2.314177 +0.756780 +0.894035

```
16
T1minBir (19)
C -1.732963 +0.271850 -1.153829
C -1.504024 +0.415742 +0.210034
C -0.698581 -0.119642 -2.037461
C +0.575783 -0.370327 -1.518398
C -0.207463 +0.160748 +0.744656
C +0.842269 -0.237796 -0.140198
N -0.069859 +0.343451 +2.122773
O +1.133515 +0.082348 +2.752883
O -0.986787 +0.722105 +2.856217
C +2.213931 -0.521874 +0.341375
O +3.177902 -0.857965 -0.290092
H +1.774567 -0.171718 +2.034916
H +1.400111 -0.676137 -2.170632
H -0.888460 -0.228070 -3.108759
H -2.737786 +0.468440 -1.542363
H -2.292154 +0.718846 +0.898878
```
